# The Mohawk homeobox gene represents a marker and osteo-inhibitory factor in calvarial suture osteoprogenitor cells

**DOI:** 10.1038/s41419-024-06813-4

**Published:** 2024-06-17

**Authors:** Yiyun Wang, Qizhi Qin, Ziyi Wang, Stefano Negri, Takashi Sono, Robert J. Tower, Zhao Li, Xin Xing, Mary Archer, Neelima Thottappillil, Manyu Zhu, Allister Suarez, Deok-Ho Kim, Tyler Harvey, Chen-Ming Fan, Aaron W. James

**Affiliations:** 1 Department of Pathology, Johns Hopkins University, Baltimore, MD 21205 USA; 2 Orthopaedic and Trauma Surgery Unit, Department of Surgery, Dentistry, Paediatrics and Gynaecology of the University of Verona, 37134 Verona, Italy; 3 Department of Biomedical Engineering, Johns Hopkins University, Baltimore, MD USA; 4 Department of Embryology, Carnegie Institution of Washington, Johns Hopkins University, Baltimore, MD 21218 USA

**Keywords:** Cell biology, Biomarkers

## Abstract

The regeneration of the mammalian skeleton’s craniofacial bones necessitates the action of intrinsic and extrinsic inductive factors from multiple cell types, which function hierarchically and temporally to control the differentiation of osteogenic progenitors. Single-cell transcriptomics of developing mouse calvarial suture recently identified a suture mesenchymal progenitor population with previously unappreciated tendon- or ligament-associated gene expression profile. Here, we developed a Mohawk homeobox (*Mkx*^*CG*^*; R26R*^*tdT*^) reporter mouse and demonstrated that this reporter identifies an adult calvarial suture resident cell population that gives rise to calvarial osteoblasts and osteocytes during homeostatic conditions. Single-cell RNA sequencing (scRNA-Seq) data reveal that *Mkx*^+^ suture cells display a progenitor-like phenotype with expression of teno-ligamentous genes. Bone injury with *Mkx*^*+*^ cell ablation showed delayed bone healing. Remarkably, *Mkx* gene played a critical role as an osteo-inhibitory factor in calvarial suture cells, as knockdown or knockout resulted in increased osteogenic differentiation. Localized deletion of *Mkx* in vivo also resulted in robustly increased calvarial defect repair. We further showed that mechanical stretch dynamically regulates *Mkx* expression, in turn regulating calvarial cell osteogenesis. Together, we define *Mkx*^+^ cells within the suture mesenchyme as a progenitor population for adult craniofacial bone repair, and *Mkx* acts as a mechanoresponsive gene to prevent osteogenic differentiation within the stem cell niche.

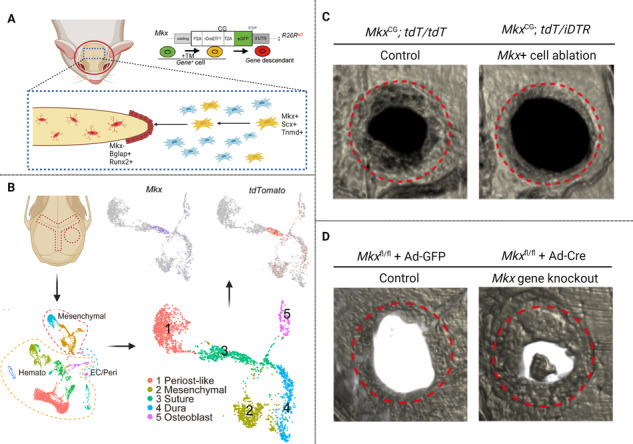

## Introduction

The skeletal stem cell niche – a reservoir of progenitor cells that form and replenish bone throughout the lifespan – requires both intrinsic and extrinsic regulatory factors for its maintenance. The calvarial suture mesenchyme represents one such location of skeletal stem cells [1]. The calvaria is formed via intramembranous ossification, and calvarial suture mesenchyme is a fibrous joint that sits between bones of the skull, holding them together. Mechanical tension, whether the result of intrinsic growth or externally applied force, models the suture and is one determinant of the extent of osteoblastogenesis and osteoblast activity [2, 3]. Key cells and/or signaling factors must counterbalance these osteoinductive pressures to preserve the integrity of the calvarial suture niche. At least four markers of resident suture mesenchymal stem cells subpopulations have been identified in the calvarial suture, including *Gli1*^+^, *Axin2*^+^, *Prrx1*^+^, and *Ctsk*^+^ cells [1, 4–8]. Although these four calvarial suture cell subsets share some common stem cell properties, differences exist between these cell subpopulations. For example, *Gli1*^+^ cells are more inclined to differentiate into osteoblasts and chondrocytes [9, 10], while *Axin2*^+^ cells possess self-renew capability and contribute to stem cell niche maintenance [4]. *Prx1*^+^ cells are identified in the calvaria and axial skeleton, and decrease in number with age [5]. *Ctsk*^+^ cells are predominantly found in the suture mesenchyme and regulate the process of intramembranous ossification [6]. Developing mouse calvarial sutures have been recently examined using single-cell transcriptomics, which further revealed the heterogeneity of the calvarial suture niche. A population of suture-associated mesenchymal progenitor cells emerged with tendon- or ligament-associated gene expression profile, but without characterization [11, 12].

Among teno-ligamentous genes is the transcription factor Mohawk (*Mkx*), an IRX-family homeobox protein involved in vertebrate developmental patterning and critical for tenogenesis [13–15]. *Mkx* has also been implicated in homeostatic maintenance of the teno-ligamentous microenvironments, such as the periodontal ligament [16] and Achilles tendon [17]. *Mkx* has an important mechanotransductive role in tendon and tenocytes [15, 18, 19], but such a role in other tissues has not been explored. Moreover, recent work in mouse and rat show that *Mkx*-null animals contain pathologic ossification within tendons [15, 17], suggesting *Mkx* regulates osteogenic differentiation, at least in pathologic contexts outside of bone tissue.

In this study, we used mouse models and single-cell transcriptomics to investigate the role of *Mkx*^+^ calvarial suture cells. We found that *Mkx*^+^ cells represent a subpopulation of mesenchymal progenitors that differentiate into osteoblasts and osteocytes during homeostatic conditions and during calvarial defect repair. In addition, we found that the *Mkx* gene functions as an osteogenic inhibitor in osteoprogenitor cells. Depleting the *Mkx* gene resulted in increased osteoblast differentiation both in vitro and in vivo. Finally, we observed that *Mkx* is a mechanically responsive gene and that mechanical stress reduced *Mkx* expression while inducing osteogenic differentiation in cranial suture cells.

## Results

### Identification of a tendo- and ligamentocyte-like calvarial suture osteoprogenitor cell

**Fig. 1:**
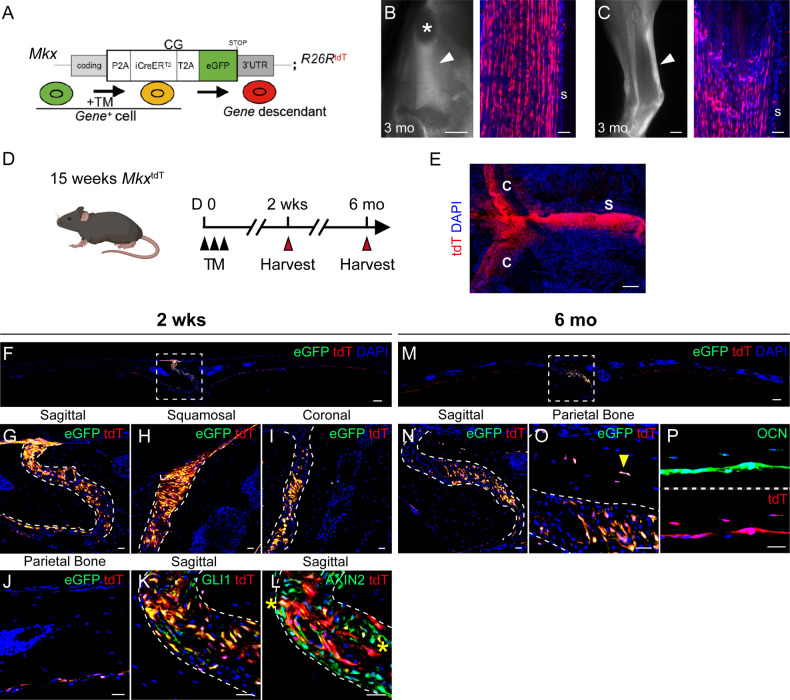
*Mkx*^+^ cells populate the calvarial suture mesenchyme and contribute to calvarial bone turnover. **A** Diagrams of the *Mkx*^*CG*^ driver. *Mkx*-expressing cells are labeled with GFP. Following tamoxifen (TM) administration, *Mkx*-expressing cells will be permanently labeled with TdTomato (tdT). **B** Whole-mount fluorescent image (left) and histology of tdT reporter activity (right) in patellar tendon of a 3-month old *Mkx*^*CG*^*;R*^*tdT*^ mice post TM injection. Arrowhead: patellar tendon. s: tendon sheath. Asterisk: patella. GFP not shown. **C** Whole-mount fluorescent image (left) and histology of tdT reporter activity (right) in Achilles tendon of a 3-month old *Mkx*^*CG*^*;R26R*^*tdT*^ (denoted as *Mkx*^tdT^) mice post TM injection. Arrowhead: Achilles tendon. s: tendon sheath. GFP not shown. **D** Schematic of TM administration in 15 weeks-old mice, with sample collection at 2 wks and 6 mo. **E** Top-down whole-mount image of calvarial bones 2 wks after TM injection. C: coronal suture and S: sagittal suture. GFP not shown. **F**–**J** Representative images of *Mkx* reporter activity within **F** tile scan of parietal bone, **G** sagittal suture, **H** squamosal suture, **I** coronal suture, and **J** Dura at 2 weeks after TM administration. **K**, **L** Colocalization of *Mkx* reporter activity with **K** GLI1 and **L** AXIN2 immunostaining. Reporter activity appears red, immunoreactivity appears green, and colocalization appears yellow (indicated with asterisk). GFP reporter activity not shown. **M**–**P** Representative images of *Mkx* reporter activity within **M** Tile scan of parietal bone, **N** sagittal suture, and **O** suture adjacent osteocytes at 6 months after TM administration. Yellow arrowheads indicate reporter positive osteocyte. **P** OCN immunostaining and *Mkx* reporter activity at 6 months after TM administration. GFP reporter activity not shown. n = 4 mice per time point. Scale bars length: **B**, **C** (left): 1000 μm. **F**, **M**: 100 μm. **B**, **C** (right), **G**–**L** and **N**–**P**: 20 μm.

Previous studies reported a ‘tenocyte-like’ or ligamentocyte-like’ gene signature within suture mesenchyme of the late mouse embryo, including expression of *Mohawk* (*Mkx*) [11, 12]. To further examine this, we engineered *Mkx* reporter mice *Mkx*^CG^; R26R^tdT^ (denoted as *Mkx*^tdT^): A CreER^T2^ and an eGFP reporter are inserted at the *Mkx* locus (*Mkx*^CG^) to identify *Mkx*^+^ (eGFP^+^) cells and to indelibly mark the *Mkx* lineage with a Cre-reporter R26R^tandem-Tomato^ (R26R^tdT^) by tamoxifen (TM) injection intraperitoneally (Fig. 1A, Supplementary Fig. S1). The *Mkx* reporter activity was initially verified in patellar and Achilles tendons (Fig. 1B, C). We then examined *Mkx* reporter activity in calvarial sutures at 2 weeks and 6 months after TM administration in adult mice (Fig. 1D–P). In whole-mount preparations, we observed robust *Mkx* reporter activity within all calvarial sutures (Fig. 1E). *Mkx* reporter activity within calvarial sutures was further confirmed in histological sections, including sagittal, squamosal, and coronal sutures (Fig. 1F–I**)**. In addition, we confirmed that endogenous MKX overlaps with reporter activity via immunofluorescence staining within the sagittal suture **(**Supplementary Fig. S2). A small amount of *Mkx* reporter activity was observed in dura mater underlying the parietal bone (Fig. 1J). Moreover, immunostaining revealed that *Mkx* reporter activity overlapped with other suture cell markers, including GLI1 and AXIN2 (Fig. 1K, L). Immunostaining revealed that GLI1 positive cells were located primarily in the center of the suture, while AXIN2 positive cells were more widely distributed toward the calvarial bone edges (Fig. 1K, L). Long-term chasing (up to 6 months) showed that suture resident *Mkx*^*+*^ cells contribute to calvarial bone turnover, becoming osteoblasts and osteocytes over time (Fig. 1M–P). The existence of *Mkx*^+^ cells within midline calvarial sutures was further verified by spatial transcriptomic analysis of sagittal sutures, in which both *Mkx* and other tendon markers were found in early SpatialTime, reflecting expression within the calvarial midline (Supplementary Fig. S3) [20]. These findings together suggest a tendon-like suture resident *Mkx*^+^ cells involved in calvarial bone turnover.

### Single-cell analysis of *Mkx*-expressing suture mesenchyme

**Fig. 2:**
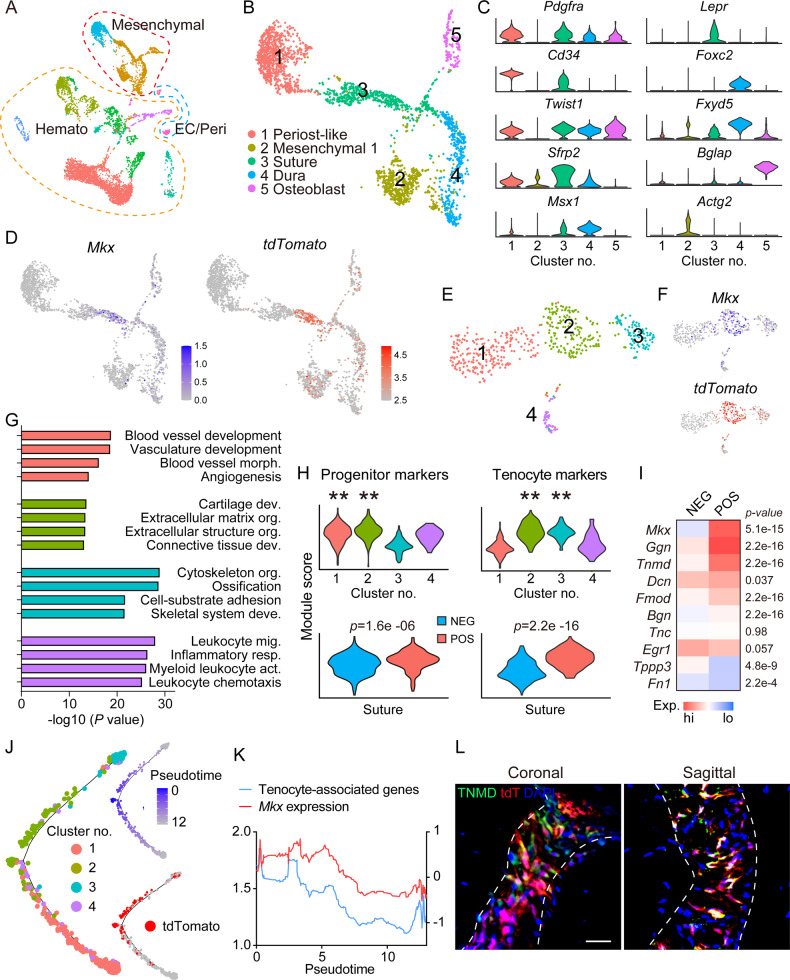
Single-cell RNA sequencing of the intact calvaria within *Mkx* reporter animals. *Mkx*^tdT^ animals (male and female, 8-week old, n = 3) were administered TM for three consecutive days, and subjected to sacrifice after 5 days from the last dosage. The frontal and parietal bone including coronal and sagittal sutures was micro-dissected, dissociated, and subjected to scRNA-seq. **A** Uniform Manifold Approximation and Projection (UMAP) plot of total cells isolated from intact calvaria. Hemato hematopoietic cell, EC endothelial cell, Peri pericyte. **B** UMAP of mesenchymal lineage cells. **C** Violin plots of marker gene expression for subclusters. **D** UMAP plot of *Mkx* expression and tdTomato reporter activity. **E** UMAP plot of subclusters of suture cells only. **F** UMAP plot of *Mkx* expression and tdTomato reporter activity across subclusters. **G** Selected GO terms and KEGG pathways enriched in each subcluster. **H** Module score of progenitor cell markers and tenocyte markers across 4 subclusters (top) and in *Mkx*^−^ and *Mkx*^+^ cells from all subclusters (bottom). The tdTomato expression >2.5 was considered as *Mkx* positive. **I** Heatmap showing selected tenocyte-associated genes among tdT^−^ and tdT^+^ cells in suture cells. **J** Trajectory analyses of cell clusters showing the distribution of identified 4 subclusters and pseudotemporal cell ordering along tdTomato trajectories. **K** Tenocyte-associated genes and *Mkx* gene expression over pseudotime. **L** Immunostaining of TNMD and tdTomato reporter activity in coronal and sagittal suture (n = 4). GFP reporter activity not shown. Scale bar: 20 μm.

To gain insight into *Mkx*^+^ suture cells, single-cell RNA sequencing (scRNA-Seq) profiling of intact calvarial bone was applied to *Mkx*^*tdT*^ animals. Of 8231 cells, unsupervised clustering identified mesenchymal (2473 cells), hematopoietic (5577 cells), and endothelial/pericyte (181 cells) clusters, based on characteristic gene markers (Fig. 2A, Supplementary Fig. S4, Supplementary File S1). Focused analysis of the mesenchymal cell cluster yielded five subclusters, assigned to periosteal-like (expressing *Pdgfra* and *Cd34*), mesenchymal 1 (expressing *Actg2* and *Msx1*), suture (expressing *Lepr* and *Sfrp2*), dura (expressing *Foxc2* and *Fxyd5*), and osteoblast subclusters (expressing *Bglap* and *Ibsp*. Figure 2B, C, Supplementary File S1). UMAP confirmed co-expression of *Mkx* and tdT reporter in 531 suture cells (Fig. 2D). We next sub-clustered the suture cells (Fig. 2E–H) and delineated four subclusters (Fig. 2E, F, Supplementary File S1). Isolated analysis of these four subclusters showed that DEGs enriched for distinct GO terms are associated with cartilage development and connective tissue development (subcluster No. 2), as well as cytoskeleton organization and ossification (subcluster No. 3), whereas blood vessel development and blood vessel morphogenesis were enriched in subcluster No. 1, and inflammatory response and leukocyte chemotaxis were enriched in subcluster No. 4 (Fig. 2G, see Supplementary Tables S1–S4 for top GO terms expressed among each category). Next, suture cells were stratified by *Mkx* reporter expression. Progenitor cell markers were expressed by all 4 subclusters of suture cells, but enriched in subclusters 1 and 2 (*p* < 0.01), while tenocyte markers were enriched in subclusters 2 and 3 (Fig. 2H, *p* < 0.01). When suture cells were subdivided into *Mkx*^−^ and *Mkx*^+^ subsets, *Mkx*^+^ suture cells showed a clear enrichment in both progenitor and tenocyte gene profile (Fig. 2H, *p* = 1.6e-6, *p* = 2.2e-16, respectively). *Mkx*^+^ suture cells showed enrichment for a host of genes closely associated with tenocytes (Fig. 2H, Supplementary File S2). We thus analyzed the tenocyte-related gene profile in *Mkx*^+^ cells. Heatmap showed higher expression of Tenomodulin (*Tnmd, p* = 2.2e-16), Fibromodulin (*Fmod, p* = 2.2e-16), and Biglycan (*Bgn, p* = 2.2e-16) in *Mkx*^+^ cells (Fig. 2I). Pseudotime analysis showed pseudotemporal cell distribution along tdT trajectories (Fig. 2J), and tenocyte-associated genes expression was paralleled with *Mkx* gene expression (Fig. 2K). Furthermore, the expression of tenocyte-associated gene, *Tnmd*, was verified by immunostaining in coronal and sagittal sutures (Fig. 2L). In sum, our scRNA-Seq data and immunostaining suggested both a dual ‘tenocyte-like’ and ‘progenitor cell-like’ status for *Mkx*-expressing cells within calvarial suture mesenchyme.

### *Mkx*^+^ cells in the suture mesenchyme contribute to defect repair

**Fig. 3:**
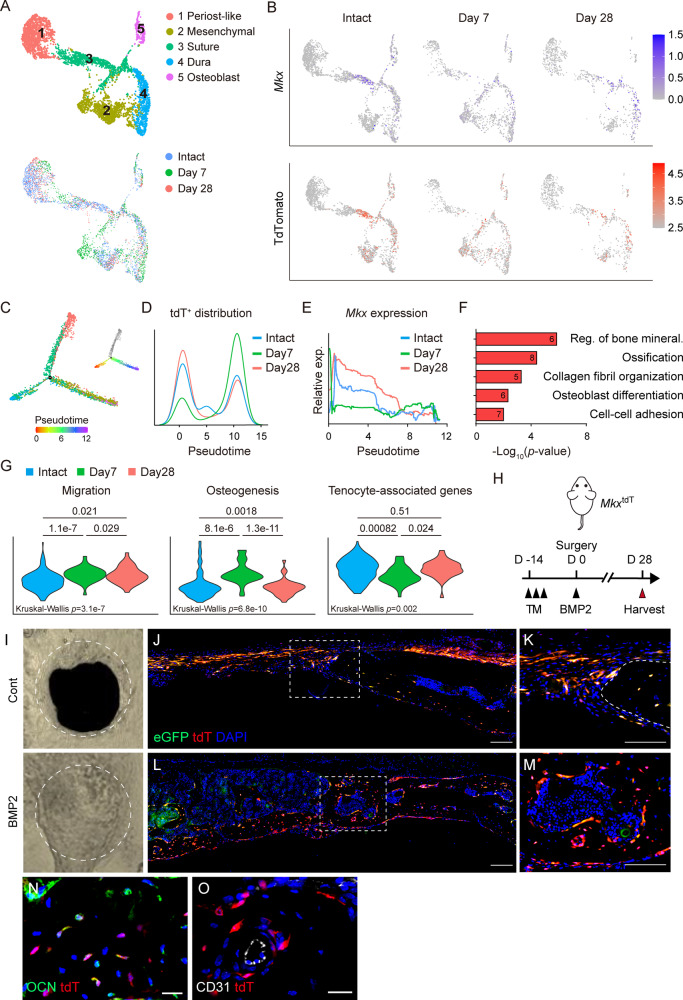
*Mkx*^+^ cells contribute to calvarial bone repair. **A**–**H** Single-cell RNA sequencing of calvarial defects within *Mkx*^tdT^ reporter mice in relation to intact calvaria. **A** UMAP plot of cell clusters all time points (top) and across time points after defect creation (bottom). N = 3 mice/time point. Intact: 8321 cells (data also shown in Fig. 2), Day 7: 6699 cells, Day 28: 7065 cells. **B** UMAP plot of *Mkx* gene expression (top) and tdTomato reporter activity (bottom) across time. **C** Pseudotemporal cell ordering along differentiation trajectories. Pseudotime is depicted from red to purple. The tdT negative state (appearing in gray) was not included for downstream analyses. **D** *Mkx*^tdT^ cell distribution over pseudotime. **E** *Mkx* gene expression over pseudotime. **F** GO terms enriched in *Mkx*^tdT^ cells on day 7. **G** Module score of cell migration, osteogenesis, and tenocyte-associated genes in *Mkx*^tdT^ cells across time points. A Kruskal–Wallis test with Dunn’s multiple comparison was used to determine differences between groups. **H** Schematic for calvarial bone defect with or without BMP2 treatment in *Mkx*^tdT^ reporter mice. **I** μCT reconstructions of the defect area without or with BMP2 treatment. The margins of original defect are indicated by dashed white. **J**–**M** Representative tile scans and high-magnification images of *Mkx* reporter activity in the calvarial defect site **J**, **K** without or **L**, **M** with BMP2 treatment at 28 d post-defect. Reporter activity appears red and green, nuclear counterstain appears blue. Dashed white lines at high-magnification images indicate healing bone edge. Scale bar: 100 μm (**J**, **L**) or 20 μm (**K**, **M**). **N** Osteocalcin (OCN) immunostaining and **O** CD31 immunostaining within the healing area on day 28 after defect. GFP reporter activity not shown in (**M**, **O**). Scale bar: 20 μm. n = 3 for (**I**–**O**).

To test whether *Mkx*^+^ suture cells can function as progenitors to repair calvaria bone directly, we subjected *Mkx*^tdT^ animals to frontal bone defects and performed scRNA-Seq profiling of the defect area at 7 and 28 d after bone defect. Again, scRNA-Seq revealed that five mesenchymal lineage cell populations were represented before and after calvarial defect, with 2429 mesenchymal cells assayed in the intact calvarial group, and 1288 and 942 cells within the day 7 and day 28 samples (Fig. 3A, Supplementary Fig. S4). UMAP confirmed that the *Mkx* and tdT co-expression in the suture cluster within the intact calvarial sample, as well as day 7 and day 28 after bone defect (Fig. 3B). Among *Mkx*^*+*^ cells, trajectory analysis of differentiation identified the distribution of tdT^+^ cells to be shifted over pseudotime after defect (Fig. 3C, D, Supplementary Fig. S5**)**. tdT^+^ cells were enriched in early pseudotime (x-axis <5) among cells from the intact calvaria (Fig. 3D), shifted toward later pseudotime (>5) acutely after calvaria defect (d 7), and then shifted back toward early pseudotime at later stages of healing (d 28) (Fig. 3D**)**. By contrast, *Mkx* gene expression was downregulated over pseudotime throughout the course of the healing process, and *Mkx* expression was decreased on day 7 and restored on day 28 after defect (Fig. 3E). Consistent with pseudotime analysis, GO term analysis among *Mkx*^*+*^ cells identified biological process related to bone mineralization, ossification, and osteoblast differentiation on day 7 after defect creation (Fig. 3F, Supplementary Table S5). To better understand the functional characterization of *Mkx*^+^ cells during bone repair, the *Mkx*^+^ cluster was further analyzed by cellular function-related gene expression, shown by cumulative gene module scoring. Module scores of *Mkx*^*+*^ cells revealed dynamic changes across time after defect. For example, an increase in module scores related to cellular migration and osteogenesis were observed on day 7 (Fig. 3G, Supplementary File S3). Conversely, reduced expression of tenocyte-associated genes was found at the same time point (Fig. 3G). To further investigate the correlation between *Mkx* and bone healing, *Mkx*^tdT^ animals were subjected to calvarial defect and treated with either vehicle control or BMP2 (Fig. 3H). BMP2 was used as a commonly used cytokine to accentuate bone healing. MicroCT reconstruction confirmed complete healing with BMP2 treatment (Fig. 3I). An immunofluorescence tile scan of the defect edge showed tdT reporter activity in the newly formed bone (Fig. 3J–M, Supplementary Fig. S6). Moreover, many bone-lining osteoblasts and bone-entombed osteocytes of the new-forming bone were tdT^+^, and osteocalcin (OCN) expression overlapped with tdT reporter activity within osteocytes (Fig. 3N), while no overlap with CD31^+^ endothelium was seen (Fig. 3O). Those findings indicated that *Mkx*^*+*^ cells in the suture mesenchyme contributing to defect repair by differentiating into osteoblasts and osteocytes. In sum, the combination of scRNA-Seq and histology analysis define *Mkx*^+^ suture cells as repair-regeneration-competent osteoprogenitors in calvarial sutures.

### *Mkx+ cells* are required for calvarial bone defect repair

**Fig. 4:**
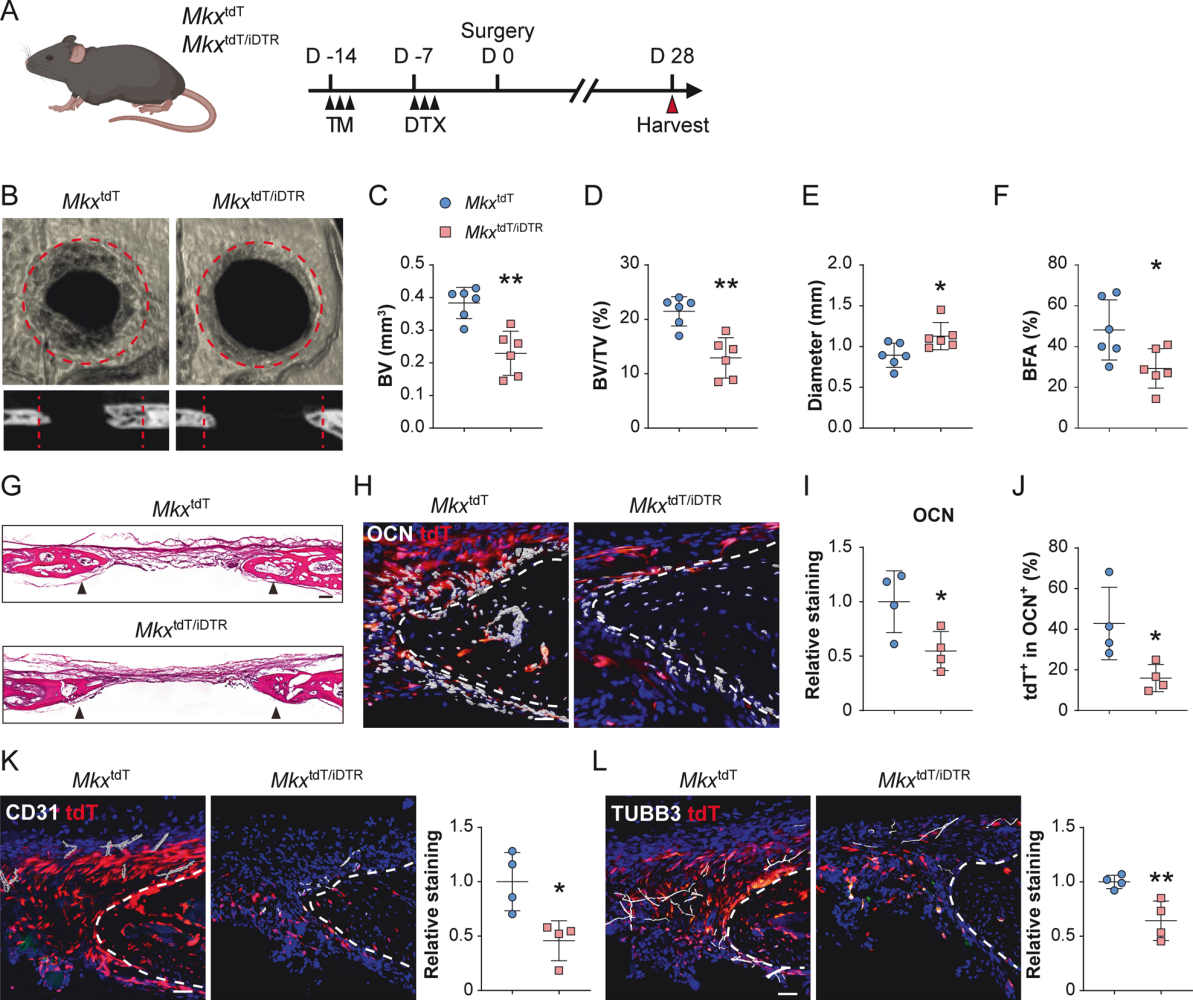
*Mkx*^+^ cell ablation leads to compromised defect repair. **A** Schematic for calvarial bone defect. *Mkx*^tdT^ or *Mkx*^tdT/iDTR^ mice were administered TM followed by local diphtheria toxin (DTX) overlying the skull, and then subjected to full-thickness frontal bone defects. Samples were harvested at day 28 post-defect. **B** μCT reconstructions of the defect site in a top-down view (above) and sagittal cross-sectional images (below). Margins of original defect are indicated by dashed red lines. **C**–**G** μCT quantification of bone healing among *Mkx*^tdT^ and *Mkx*^tdT/iDTR^ mice, including **C** bone volume (BV), **D** bone volume/tissue volume (BV/TV), **E** residual defect diameter, and **F** bone fractional area (BFA). **G** Hematoxylin and eosin (H&E) staining of coronal cross sections of the healing defect site from *Mkx*^tdT^ or *Mkx*^tdT/iDTR^ mice, d 28 after defect. Black arrowheads indicate original defect sites. Scale bar: 100 μm. **H**–**J** Immunostaining of OCN at the defect edge (**H**) and quantification of OCN (**I**) and percentage of *Mkx*^tdT^ cells among OCN^+^ cells within the defect site (**J**). GFP reporter activity not shown. **K** Immunostaining of CD31^+^ blood vessels at the defect edge from *Mkx*^tdT^ and *Mkx*^tdT/iDTR^ mice (left) and quantification (right). **L** Immunostaining of TUBB3^+^ (Beta III tubulin) nerve fibers at the defect edge from *Mkx*^tdT^ and *Mkx*^tdT/iDTR^ mice (left) and quantification (right). White dashed lines indicate healing bone edges. Scale bar: 100 μm. Dots in scatterplots represent values from individual measurement, whereas mean and 1 SD are indicated by crosshairs and whiskers. Relative staining: individual value was normalized to mean fluorescence intensity of control group (*Mkx*^tdT^). *p < 0.05; ** *p* < 0.01. A two-tailed Student t-test was used for all comparisons.

We next generated *Mkx*^tdT/iDTR^ mice by crossbreeding *Mkx*^tdT^ mice with previously validated iDTR mice, in which Cre-induced expression of diphtheria toxin receptor (DTR) renders cells susceptible to ablation following diphtheria toxin (DTX) treatment. Next, *Mkx*^+^ cell ablation was achieved by local DTX administration. Lineage tracing of *Mkx*-expressing cells was based on tamoxifen administration starting 14 days prior calvarial defect (Fig. 4A). Next, frontal bone healing was assessed following *Mkx*^+^ cell ablation in *Mkx*^tdT^ and *Mkx*^tdT/iDTR^ mice over a 28-day period (Fig. 4B–L). MicroCT reconstructions and cross-sectional images demonstrated impaired re-ossification among *Mkx*^tdT/iDTR^ animals compared to *Mkx*^tdT^ animals (Fig. 4B). Quantitative microCT metrics of bone healing were reduced among *Mkx*^tdT/iDTR^ mice, including bone volume (BV, 40.2% reduction, Fig. 4C), fractional BV (BV/tissue volume (TV), 39.9% reduction, Fig. 4D), mean diameter of the bone defect area (36.3% increase, Fig. 4E), and bone fractional area (BFA, 39.2% reduction, Fig. 4F). Hematoxylin and eosin (H&E) staining confirmed a notable impairment of healing between bony fronts in *Mkx*^tdT/iDTR^ mice (Fig. 4G, black arrowheads). Validation for *Mkx*-expressing cell was performed using OCN immunohistochemistry and tdT reporter activity on calvarial defect sites of *Mkx*^tdT^ and *Mkx*^tdT/iDTR^ mice (Fig. 4H–J). Following defect, a clear reduction of OCN immunostaining was detected along the outer edge of the defect site among *Mkx*^tdT/iDTR^ mice (Fig. 4H, I. 45.3% reduction). Furthermore, within the OCN^+^ cells, a significant reduction in tdT reporter activity was observed among *Mkx*^tdT/iDTR^ animals compared to *Mkx*^tdT^ animals (Fig. 4J, 62.7% reduction). As indicated by our previous study, coupled innervation and angiogenesis play important roles in calvarial bone repair [21]. The reduction in bone healing in *Mkx*^tdT/iDTR^ animals was also associated with reduced angiogenesis and innervation at the healing edge, shown as decreased CD31^+^ blood vessels (54.3% reduction) and Tubb3^+^ nerves (35.8% reduction) (Fig. 4K, L). Altogether, our data suggesting the *Mkx*^+^ cells represent a pool of osteoprogenitor cells within the murine calvaria necessary for proper bone defect repair.

### *Mkx* functions as an osteogenic inhibitor within calvarial defect

**Fig. 5:**
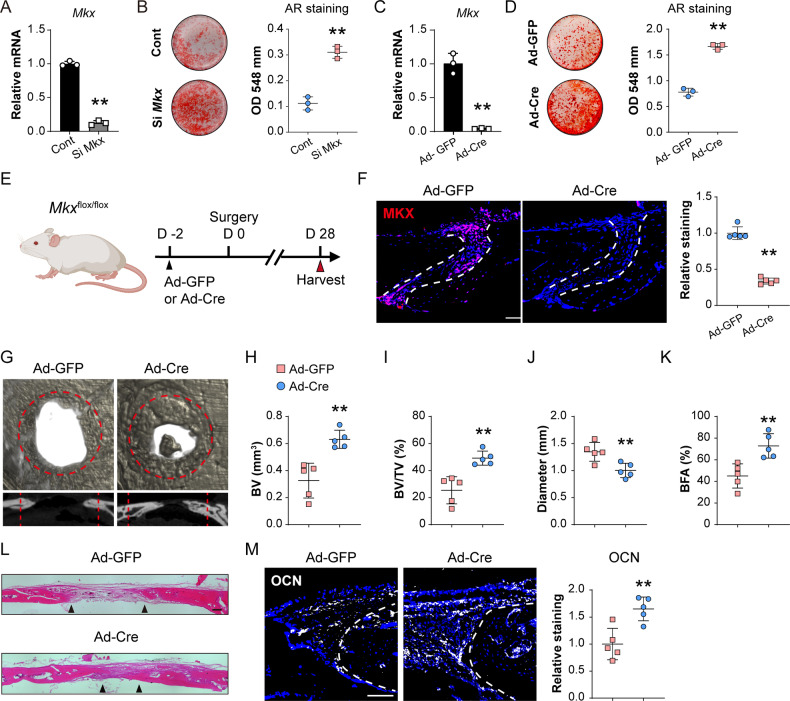
*Mkx* gene knockdown or knockout induces osteogenic differentiation and calvarial bone repair. **A**, **B** siRNA mediated knockdown of *Mkx* within calvarial suture cells. **A** Confirmation of siRNA mediated *Mkx* knockdown efficiency by qPCR, 48 h after siRNA treatment. **B** Representative Alizarin red staining at d 10 of osteogenic differentiation (left) and quantification (right). **C**, **D** Ad-Cre mediated knockout of *Mkx* within *Mkx*^fl/fl^ calvarial suture cells. **C** Confirmation of Ad-Cre mediated *Mkx* knockout efficiency by qPCR, 48 h after Ad-Cre treatment. **D** Representative Alizarin red staining at d 10 of osteogenic differentiation (left) and quantification (right). **E** Schematic of calvarial bone defect. *Mkx*^flox^ mice were locally administered Ad-Cre or Ad-GFP directly overlying the skull, and thereafter subjected to calvarial defect creation. Samples were harvested at d 28 post-defect. **F** *Mkx* gene knockout efficiency tested by immunostaining (left) and quantification (right) within the sagittal suture of *Mkx*^flox^ mice 28 d after viral injection. **G** μCT reconstructions of the defect site in a top-down view (above) and sagittal cross-sectional images (below). Analysis performed at d 28 post-defect. Margins of original defect are indicated by dashed red lines. **H**–**K** μCT quantification of bone healing among control and Ad-Cre treated mice, including **H** bone volume (BV), **I** bone volume/tissue volume (BV/TV), **J** residual defect diameter, and **K** bone fractional area (BFA). **L** H&E staining of coronal cross section of the healing defect site from control and Ad-Cre treated mice, d 28 after defect. Black arrowheads indicate healing bone edges. Scale bar: 100 μm. **M** Immunostaining of OCN at the defect edge (left) and quantification of OCN (right). Dots in scatterplots represent values from individual measurement, whereas mean and 1 SD are indicated by crosshairs and whiskers. *p < 0.05; **p < 0.01. A two-tailed Student t-test was used for all comparisons.

In tenocytes, *Mkx* has been implicated as an inhibitor of osteogenic differentiation. To further understand the function of *Mkx* gene in calvarial suture cell osteogenic differentiation, *Mkx* gene knockdown (KD) and knockout (KO) were performed in calvarial suture cells derived from *Mkx*^tdT^ and *Mkx*^fl/fl^ animals, respectively (Fig. 5A–D). First, small interference RNA induced *Mkx* KD efficiency was tested by qPCR and 87.5% KD efficiency was confirmed among *Mkx*^tdT^ suture cells (Fig. 5A). All cells then underwent osteogenic differentiation and Alizarin red (AR) staining (Fig. 5B). Significantly increased osteogenic differentiation was observed after *Mkx* gene KD in comparison to scramble control (Fig. 5B, 178.2% increase). Similar findings were observed within *Mkx*^fl/fl^ suture cells after Ad-Cre-induced *Mkx* KO (Fig. 5C, D). Here, qPCR verified a 95.9% KO efficiency (Fig. 5C) and AR staining showed a 113.9% increase in osteogenic differentiation when compared to Ad-GFP control (Fig. 5D).

To further validate *Mkx* gene function in vivo, *Mkx*^fl/fl^ animals were treated with adenovirus-expressing Cre recombinase (Ad-Cre) within the defect site, while *Mkx*^fl/fl^ littermates received Ad-GFP as a vector control (Fig. 5E) using previously established protocols [21]. Ad-Cre-induced recombination in *Mkx*^fl/fl^ animals was validated by MKX immunostaining within sagittal suture, and a 66.7% reduction of MKX immunostaining was observed (Fig. 5F). Next, animals with or without local *Mkx* gene deletion were subjected to frontal bone defect, with μCT assessments performed at 28 days post-defect (Fig. 5G–L). μCT reconstructions demonstrated a significant improvement in bone repair among Ad-Cre-treated *Mkx*^fl/fl^ animals compared to Ad-GFP-treated controls (Fig. 5G), which was validated by μCT analysis. Ad-Cre-treated animals showed a significant increase in BV (93.7% increase, Fig. 5H), BV/TV (92.7% increase, Fig. 5I), mean diameter of the bone defect area (25.6% reduction, Fig. 5J), and BFA (61.7% increase, Fig. 5K). Again, H&E staining confirmed a notable improvement in defect healing between bony fronts in Ad-Cre-treated animals (Fig. 5L). The increased ossification at the healing edge was further validated by OCN immunostaining, and a 65.1% increase was confirmed (Fig. 5M). Together, our results imply that not only *Mkx*^+^ calvarial suture cells are engaged in calvarial bone repair, but *the Mkx* gene also plays a key role in these progenitor cells to restrain osteogenic differentiation and inhibits bone defect repair.

### RNA sequencing reveals *Mkx* gene regulating mesenchymal progenitor osteogenic differentiation and response to mechanical stimulus

**Fig. 6:**
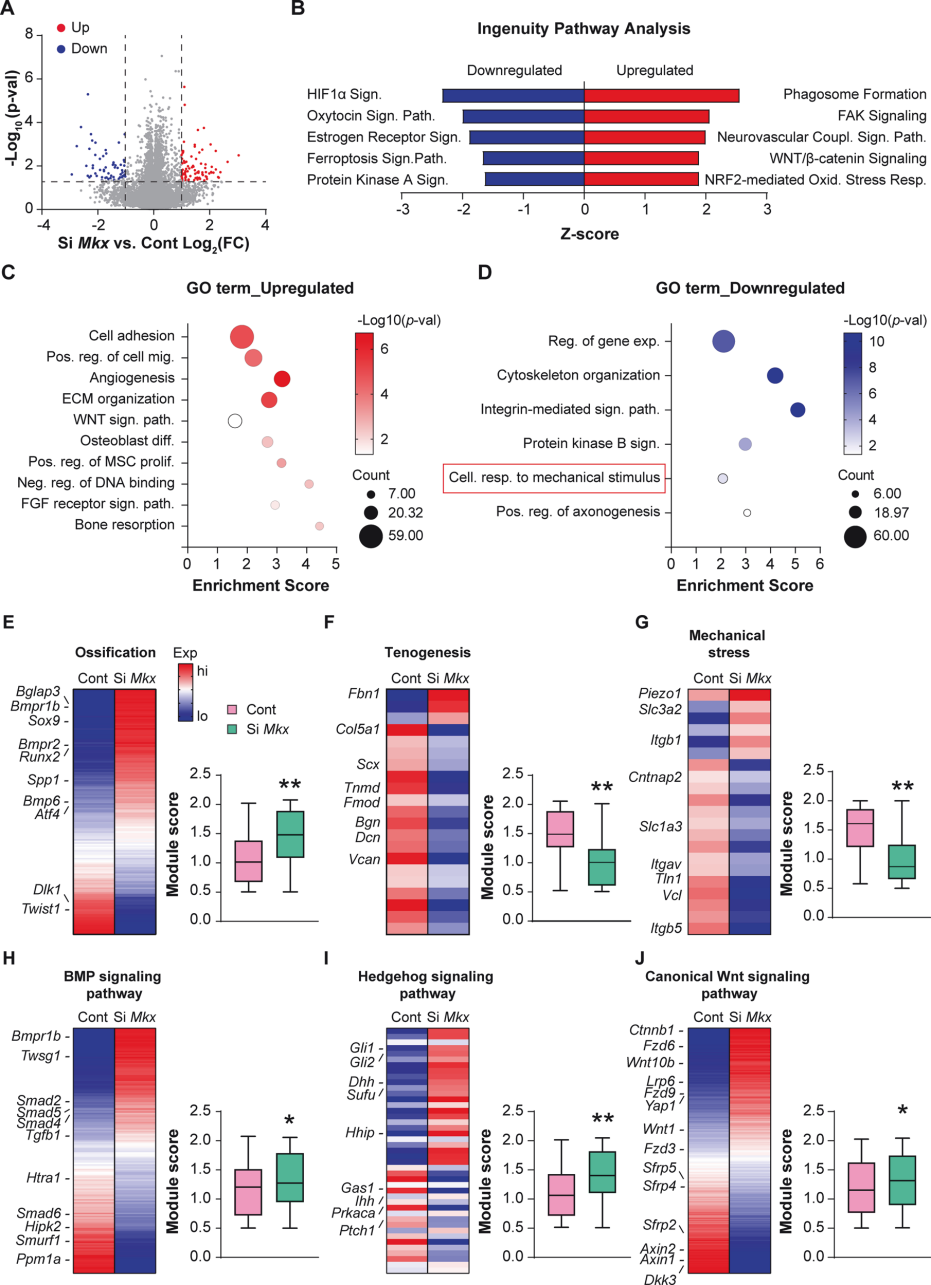
Total RNA sequencing reveals signaling alterations after *Mkx* knockdown in calvarial suture cells. **A** Volcano plot summarizing differentially expressed genes (DEGs) (FDR < 0.05). DEGs with fold change (Log2FC) over 1 and p-value less than 0.05 were colored red or blue. Red dots are significantly upregulated DEGs after *Mkx* KD, while blue dots are significantly downregulated DEGs in comparison to scramble control. N = 3 biological replicates. **B** Ingenuity Pathway Analysis (IPA) showing top canonical pathways upregulated and downregulate among *Mkx* KD cells in comparison to control. **C**, **D** Bubble plot showing GO enrichment analysis identified representative pathways that were upregulated (**C**) or downregulated (**D**) among *Mkx* KD cells. **E**–**J** Heatmaps and corresponding module scores among *Mkx* KD cells versus control in key biological processes and signaling pathways, including **E** Ossification, **F** Tenogenesis, **G** Mechanical stress, **H** BMP signaling, **I** Hedgehog signaling, and **J** Canonical Wnt signaling. Gene module scores are shown as a boxplot with center line as the median, box limits as upper and lower quartiles of the modulus score. *p < 0.05; **p < 0.01. A two-tailed Student t-test was used for all comparisons.

To characterize the transcriptomic landscape following *Mkx* gene deletion in suture mesenchymal cells, total RNA-seq analysis was next performed. Differential expression and principal component analysis (PCA) revealed that the *Mkx* KD groups exhibited significantly different expression patterns compared to siRNA control (Supplementary Fig. S7, by PCA increased dispersion was observed among *Mkx* KD samples which may be caused by the non-specific effect of siRNA). *Mkx* mRNA was among top 3 downregulated genes in KD samples (log_2_ (FC) = −6.08, *p* = 0.000163, Supplementary File S3). In total, 16,478 protein-coding genes were identified, among which 1191 genes were upregulated and 1062 downregulated in *Mkx* KD cells (*p* < 0.05, Fig. 6A). Top 200 differentially expressed genes (DEGs, *p* < 0.05, and Log_2_FC > 1 or <−1) are provided in Supplementary File S3. Subsequently, Ingenuity Pathway Analysis (IPA) and GO term analyses were performed. IPA showed upregulation of signaling pathways such as FAK and Wnt/β-catenin (Fig. 6B). Consistent with our in vitro findings, functional GO enrichment analysis showed upregulation of DEGs in biological processes related to osteoblast differentiation (Fig. 6C, Supplementary Table S6). Downregulation of the integrin-mediated signaling pathway and cell response to mechanical stimulus was observed within *Mkx* KD groups (Fig. 6D, Supplementary Table S7). Next, specific changes in gene modules after *Mkx* KD were examined in suture mesenchymal cell differentiation (Fig. 6E–J, Supplementary Tables S8 and S9, gene lists provided in Supplementary File S4). Significant increases in gene modules related to ossification were observed among *Mkx* KD cells, shown by heatmap or module scores. The osteoblast markers *Runx2* and *Spp1* were upregulated after *Mkx* KD, as well as other genes involved in osteogenic differentiation such as the BMP receptors *Bmpr1b* and *Bmpr2*. In addition, other positive regulators of ossification were also increased after *Mkx* KD, such as *Bmp6* and *Atf4* [22]. Conversely, negative regulators of osteogenic differentiation were downregulated after *Mkx* KD including *Dlk1* [23] and *Twist1* [24] (Fig. 6E). Conversely, significant reductions in gene modules related to tendon and mechanical stress (e.g. *Cntnap2*, *Tln*, and *Vcl*) were confirmed (Fig. 6F, G). Tendon markers expression including *Scx*, *Tnmd*, and *Bgn* were downregulated after *Mkx* KD (Fig. 6F). Morphogenic signaling pathways associated with osteogenesis were further analyzed, including BMP, Hedgehog, and canonical Wnt signaling pathways (Fig. 6H–J). All three pathways showed a significant increase in expression among *Mkx* KD suture cells. BMP signaling pathway genes *Bmpr1b*, *Smad4*, and *Smad5*, BMP positive regulators *Twsg1* [25] and *Smad2* [22], and *Tgfb1* [26] were all upregulated (Fig. 6H). In the hedgehog signaling pathway, hedgehog ligands *Ihh* and *Dhh*, downstream transcription factors *Gli1* and *Gli2* showed upregulation with *Mkx* KD. While expression of negative regulators of Hh signaling such as *Gas1*, *Ptch1*, and *Prkaca* [27, 28] were decreased with *Mkx* KD (Fig. 6G). For Wnt signaling pathway, Wnt signaling element *Ctnnb1*, Wnt ligands *Wnt1* and *Wnt10b*, and receptors (*Fzd3*, *Fzd6*, *Fzd9* and *Lrp6*) were all increased after *Mkx* KD (Fig. 6J). Moreover, a reduction of negative regulators of Wnt signaling (*Axin1* & *2*) and Wnt antagonists (*Sfrp2*, *Sfrp4*, *Sfrp5* and *Dkk3*) were observed among *Mkx* KD cells (Fig. 6J). These changes are consistent with prior research indicating that *Mkx* negatively regulates all three pathways [15, 17, 29]. In conclusion, RNA-seq data supported our functional experiments showing that *Mkx* gene deletion enhances osteogenesis associated with increases in several key morphogenic signaling pathways.

### Mechanical stretch reduces *Mkx* expression and increases cranial suture cells osteogenic differentiation

**Fig. 7:**
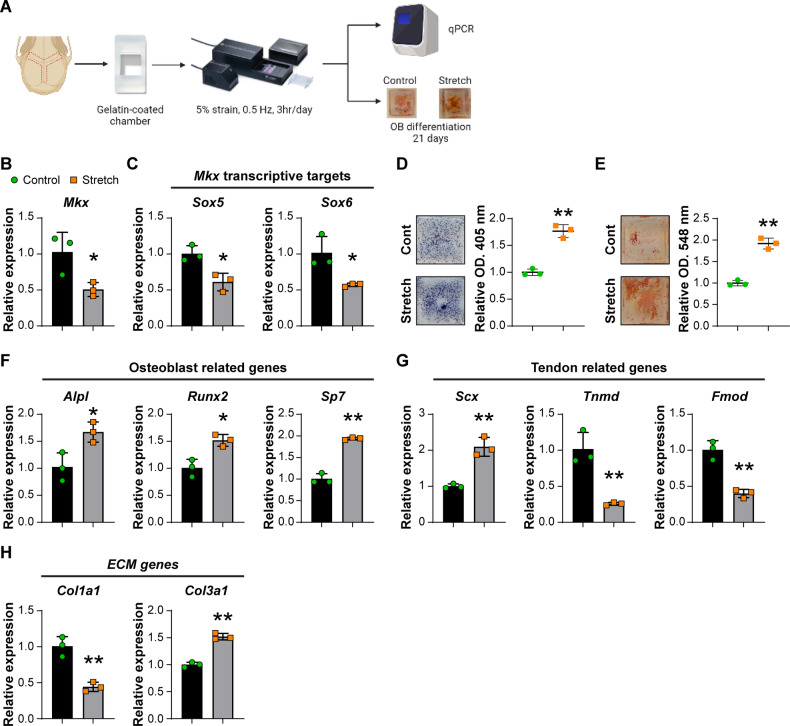
Mechanical loading of calvarial suture cells. **A** Schematic of the mechanical loading experiment. Calvarial suture cells from male 8-week-old C57BL/6J mice were seeded into gelatin-coated chambers and loaded with 5% stain at 0.5 Hz, 2 h per d for 3 d using the Cytostretcher system. Control cells were seeded in identical chambers but not subjected to biaxial strain. Gene expression was examined by qPCR 1 h after stretching or osteoblast differentiation for up to 21 d. **B** *Mkx* expression after mechanical loading by qPCR. **C** Expression of *Mkx* target genes, *Sox5* and *Sox6* by qPCR. **D** Representative images of ALP staining at 7 d (left) and statistical analysis of the staining intensity (right). **E** Representative images of alizarin red staining at 21 d (left) and quantitative analysis of staining (right). **F** Osteoblast-related genes by qPCR. **G** Expression of tendon-related genes by qPCR. **H** Expression of extracellular matrix (ECM) genes by qPCR. **B**, **C**, and **F**–**H**, data shown as mean ± 1 SD, with dots representing individual data points. **D** and **E**, individual dots in scatterplots represent values from single measurements, whereas mean and one SD are indicated by crosshairs and whiskers. All experiments were performed in triplicate replicates, with results from a single replicate shown. *p < 0.05; **p < 0.01. A two-tailed Student t-test was used for all comparisons.

During growth, a strain force is applied to the calvarial suture due to the expansion of calvaria. Multiple studies have pointed out that the mechanical stress at the calvarial suture promotes the proliferation and osteogenesis of calvarial suture cells [3, 30–32]. We next examined if *Mkx* is a mechanical-responsive gene within calvarial sutures, as has been reported in other tissues [15, 33]. For this purpose, calvarial suture cells were isolated and subjected to mechanical stretch using the Cytostretcher system (Fig. 7A). After seeding cells within the Cytostretcher chambers, a strain of 5% was applied, which is equivalent to the maximum strain at the calvarial suture during growth [34, 35]. By qPCR, we found that mechanical stimulation reduced the expression of *Mkx* in calvarial suture cells (Fig. 7B). In addition, the *Mkx* transcriptive targets *Sox5* and *Sox6* both had decreased expression after mechanical stress (Fig. 7C) [15, 36]. Osteoblast differentiation of calvarial suture cells after mechanical stretch was next performed. Seven days after osteogenic differentiation, ALP staining showed a significant increase among suture cells subjected to mechanical stretch (Fig. 7D). Likewise, 21 days after osteogenic differentiation, alizarin red staining showed more mineral deposition among mechanical stretch stimulated suture cells (Fig. 7E). Gene expression analyses 1 h after mechanical stretch showed significantly increased expression of osteoblast-related genes, such as *Alpl*, *Runx2*, and *Sp7* (Fig. 7F). We also examined the expression of tendon-related genes. Expression of *Scx* was increased but the levels of *Tnmd* and *Fmod* were decreased after mechanical stretching (Fig. 7G). Interestingly, the levels of the extracellular matrix genes, *Col1a1* and *Col3a1*, had different trends after mechanical loading, where *Col1a1* was increased and *Col3a1* was decreased (Fig. 7H). Although decreased *Col1a1* expression is not typical for cells with higher osteogenic differentiation potential, some studies in osteoblastic cells (such as MC3T3-E1 cells) have reported similar data under similar conditions [37, 38]. For example, one study by Hwang et al. revealed decreased *Col1a1* expression in primary murine calvarial osteoblasts in osteogenic media [38]. Those gene expression data suggested that *Mkx* is a mechanoresponsive gene, whose expression is negatively regulated by mechanical stretch. Loss of expression of the osteo-inhibitory gene *Mkx* is associated with enhanced osteogenesis, implicating MKX as a regulatory factor for interpreting mechanical stimuli by calvarial suture cells and regulating their osteogenic differentiation potential.

## Discussion

This study demonstrated a unique population of suture osteoprogenitor cells expressing *Mkx* with teno-ligamentous characteristics. Using a transgenic reporter mouse model, we confirmed the presence of *Mkx*^+^ cells in the calvarial sutures and that descendants of *Mkx*^+^ cells differentiate into mature osteoblasts. Depletion of this *Mkx*^+^ progenitor cell population hampered the repair of calvarial defects, yet *Mkx* functions as an osteogenic inhibitor. When *Mkx* was deleted locally, enhanced calvarial bone repair was observed. Finally, our transcriptomic and functional data provide evidence that *Mkx* acts as a mechanoresponsive gene in calvarial suture osteoprogenitor cells to link mechanical stimuli and osteogenesis within the ‘elastic’ suture niche.

With lineage tracing over time, we found that the distribution of *Mkx*^+^ calvarial suture cells also follows a similar pattern as other known progenitor populations, which reside in the suture mesenchyme. These *Mkx*^+^ cells also express cranial suture cells markers such as *Gli1* and *Axin2*. The progenies of *Mkx*^+^ cells include osteoblastic cells in the periosteum and dura and osteocytes making up the calvarial bone cortex, indicating that *Mkx*^+^ cells are osteogenic progenitors. By scRNA-seq, one unique feature of the *Mkx*^+^ calvarial suture cells are that they exhibited tendon/ligament-like phenotype, and the genetic levels of tendon-related genes are correlated with *Mkx* expression level. A previous study also identified tendon/ligament-like suture cells in the neonatal suture mesenchyme, expressing tendon-related genes such as *Tnmd*, *Scx*, and *Tnc* [11]. Thus, the *Mkx*^+^ calvarial suture cells share a similar molecular signature as tendon/ligament cells, and this teno-ligamentous feature is kept from embryonic development to adulthood in mice.

Previous studies have implicated *Mkx* in tenogenesis and the maintenance of ligament homeostasis [39, 40]. In human osteoarthritis patients, anterior cruciate ligament-derived cells showed reduced *MKX* expression, suggesting that reduced *MKX* expression is associated with the degeneration of the anterior cruciate ligament [41]. Several studies have found that in the periodontal ligament, *Mkx* is important for the homeostasis of the ligament by regulating collagen expression [16, 40]. Similarly, in *Mkx*-deficient rats, reduced *Col1a1* and *Col3a1* expression and restricted collagen fibril diameters are found in tendons [15]. Interestingly, calvarial dysmorphisms have not been reported in the *Mkx*-deficient mice and rats. However, previous studies did not focus on the calvarial phenotype of these animal models, and no studies of *Mkx’s* role in bone repair have been reported. *Mkx* also contributes to the repair of tendon injury, and in vitro overexpression of *Mkx* promotes tenogenesis by upregulating tendon-related genes [42]. Our total RNA sequencing result showed that in calvarial suture cells, reduced expression of tenocyte-related genes, *Scx*, *Tnmd*, and *Col3a1*, was resulted from knockdown of *Mkx*. Another study showed increased angiogenesis in *Mkx*^−^^/^^−^ mouse tendon, and inhibiting angiogenesis prevented HO formation in the Achilles tendon [43], which is in agreement with our data in which increased angiogenic signaling molecules are evident in *Mkx* knockdown suture cells. Interestingly, the converse findings were observed in the calvarial defect model after *Mkx*+ cell depletion, where *Mkx*^*+*^ cell depletion led to reduced blood vessel growth around the defect site. Thus, not only did the *Mkx*^+^ suture cells show a similar transcriptomic profile with tenocytes, but some similarities in signaling pathways derangement were also observable with *Mkx* deletion across distinct tissue types.

In the current study, we have identified *Mkx* as a potent osteogenic inhibitor in the context of bone repair. This too is in some agreement with findings in other tissues. For example, *Mkx*-null rats are more prone to develop heterotopic ossification in tendons due to failed tenogenesis [15]. We observed increased BMP, Wnt, and Hedgehog signaling after *Mkx* gene deletion, which might be the underlying mechanism for the increased osteogenesis, as these three signaling pathways can all promote osteogenic differentiation [44–47]. However, in tenocytes, elevated BMP signaling was not observed in *Mkx*^−^^/^^−^ mice [17]. Wnt signaling is a known suppressor of *Mkx* as overactivated Wnt signaling was correlated with inhibited *Mkx* expression in tendon-derived cells [29, 48, 49]. A study by Liu et al. showed increased Hedgehog signaling pathway in *Gli1*^+^ tendon cells in *Mkx*^−^^/^^−^ mice [17]. Thus, not only did the *Mkx*^+^ suture cells show a similar transcriptomic profile with tenocytes, but some similarities in signaling pathways derangement were also observable with Mkx deletion across distinct tissue types.

Considering the nature of tenocytes and calvarial suture, we investigated the mechanotransduction in *Mkx*-deficient suture cells. We indeed found that *Mkx* is also a mechanoresponsive gene in suture cells, where a reduced transcriptomic response to mechanical stimulus was observed after *Mkx* knockdown. Mechanical tension at the calvarial suture is generated due to the calvaria’s growth and the brain’s expansion. Such mechanical stress induces proliferation and osteogenic differentiation of the cells in the suture mesenchyme [50]. Studied signaling pathways governing the response of suture cells to mechanical stimulation, including FGF, TGF-β/BMP, and Wnt signaling [51–54]. Dysregulation of the signaling response to mechanical stress during development is related to the pathophysiology of craniofacial dysmorphisms, such as craniosynostosis. Multiple genes have been identified as osteogenic inhibitors that play important roles in maintaining the homeostasis of craniofacial development, including *Twist1, Axin2, Tcf12*, and *Noggin* [55–58]. The presence of osteogenic inhibitor genes in suture cells is thought to counteract the osteogenic differentiation induced by mechanical stress and maintain the suture [59]. Our studies implicate *Mkx* in a role similar to the aforementioned genes. Previous studies revealed that the *Mkx* levels are increased in the periosteal stem cells when mouse limbs are unloaded, but the expression of *Mkx* is increased in tenocytes with mechanical loading [15]. This indicates potentially different mechano-regulatory roles of *Mkx* for osteogenic and tenogenic cells.

In summary, *Mkx* expression typifies a calvarial suture cell with a stem/osteoprogenitor phenotype and a teno-ligamentous gene profile participating in bone turnover and repair. Furthermore, *Mkx* represents a mechanical stretch-responsive gene that functions to prevent osteogenic differentiation within the stem cell niche.

## Materials and methods

### Animals

All animal experiments were performed in accordance with approved protocols (MO16M226 & MO19M366) of the Animal Care and Use Committee (ACUC) at Johns Hopkins University (JHU). *Mkx*^P2A^^-^^iCreERT2-T2A-eGFP^; R26R^tdT/tdT^ (*Mkx*^CG^; R26R^tdT/tdT^, denoted *Mkx*^tdT^) mice were generated by the Chen-Ming Fan laboratory. *Mkx*^CG^; R26R^tdT/iDTR^ (denoted *Mkx*^tdT/iDTR^) mice were obtained by crossing *Mkx*^CG^; R26R^tdT/tdT^ mice with R26R^iDTR/iDTR^ (iDTR) mice (JAX Stock No. 007900). *Mkx*^fl/fl^ mice were donated by the Han laboratory. Mouse information are summarized in Supplementary Tables S10 and S11. Tamoxifen (TM, Sigma-Aldrich, St. Louis, MO) was injected intraperitoneally in accordance with previously validated protocols (TM: 20 mg/ml stock in flower seed oil (Sigma) and administered by intraperitoneal (i.p.) injection at 10 μl per g body weight for 3 consecutive d). Diphtheria toxin (DTX; Sigma-Aldrich) was injected subcutaneously overlying the calvaria at 4.57 mg/mL, 0.1 mL per animal 7 days after TM injection for 3 d.

### Calvarial bone defect procedures

Based on our prior methods [21, 60], calvarial bone defects were performed with male and female 8–10-week-old mice. Under general anesthesia with inhaled isoflurane (3–5% induction, 2–3% maintenance) along with subdermal injection of sustained-release buprenorphine (1.2 mg/kg SC, 72 h), the skin overlying the calvaria was prepared for surgery: hair was clipped, and skin was disinfected with povidone-iodine 5% and alcohol 70%. A 1.5 cm skin incision was made over the midline skull to expose the frontal and parietal bones. A 1.8 mm diameter, full-thickness, calvarial bone defect was created in the non-suture-associated frontal or parietal bone using a micro surgical drill and a trephine drill bit. In select experiments, a 1 mm^2^ sponge with or without 2 μg rhBMP-2 (Infuse^®^, X small kit, Medtronic, MN) was placed into the defect. Meticulous care was taken to protect the neighboring sutures and the underlying dura mater. Finally, the skin was sutured with Ethilon 5-0 suture (Ethicon Inc, Somerville, NJ) and the animals were monitored following the established postoperative protocols. Mice were euthanized at 4 wks post-bone defect creation for analysis.

### Radiographic analyses

Samples were scanned with a high-resolution μCT imaging system (SkyScan 1275; Bruker MicroCT N.V, Kontich, Belgium) with a 10 µm voxel size. The parameters of scanner were set at 1 mm of aluminum filter, X-ray voltage of 65 kVP, anode current of 153 μA, exposure time of 160–218 ms, frame averaging of 6, and rotation step of 0.3°. Then, three-dimensional images were reconstructed with image reconstruction software (NRecon, v1.7.0.4, SkyScan, Bruker). CTAn (v1.16, SkyScan, Bruker), CTVox (v3.2, SkyScan, Bruker), and CTVol (v2.0, SkyScan, Bruker) software were used to analyze 3D morphometry of images.

In order to analyze calvarial defect healing, a cylindrical volume of interest centered around each defect site was defined as the 1.8 mm in diameter and 0.7 mm in height with a threshold value of 70–255. Binary x-ray images with 2D analysis were applied to calculate Bone volume (BV) and bone volume/tissue volume (BV/TV). Bone fractional area (BFA) and defect diameter were calculated with CTVox to create a 3D rendering of the calvarial defect and measured by ImageJ software (Version 1.8.0; NIH, Bethesda, MD).

### Histology and immunohistochemistry

Tissues were harvested and fixed in 4% PFA overnight at 4 °C, then decalcified in 14% EDTA for 28 d and embedded in optimal cutting temperature compound (OCT) (Sakura, Torrance, CA). Samples were cryo-sectioned at 12 μm or 40 μm thickness. H&E staining was performed on 12 μm thick sections. For immunohistochemistry, 40 μm sections were permeabilized with 0.5% Triton-X (Sigma-Aldrich) for 20 min, blocked with 5% goat serum in PBS for 1 h at RT and incubated with primary antibodies (see Supplementary Table S12) for a summary of antibodies used overnight at 4 °C. Further, anti-Rabbit Alexa Fluor® 647-conjugated or anti-mouse Alexa Fluor® 647-conjugated secondary antibodies (1:200) were used with incubation for 2 h at RT. DAPI mounting medium (H-1500, Vector Laboratories, Burlingame, CA) was used. All sections were examined under a Zeiss 800 confocal microscope (Zeiss, Thornwood, NY) or Leica DM6 microscope (Leica Microsystems Inc, Wetzlar, Germany). Relative fluorescent staining was calculated using Imaris software (ver 9.5) (RRID: SCR_007370, Oxford Instruments plc, Tubney Woods, Abingdon, Oxon, UK), and normalized against mean fluorescence intensity of control.

### Single-cell RNA sequencing

The frontal and parietal bones including sagittal and coronal suture, with or without 1.8 mm defect (Supplementary Fig. S4A), were micro-dissected and digested with collagenase Type I/II (1 mg/mL, Worthington Biochemical Corporation, Lakewood, NJ; LS004197, LS004177) and Dispase II (2 mg/mL) for 3 × 15 min (N = 3 animals per group: intact bone, 7 d and 28 d post-bone defect). Cell fractions were collected and resuspended in red blood cell lysis buffer (RT for 10 min). Digestions were subsequently filtered through 40 μm sterile strainers. Then, cells were washed in PBS and resuspended in 0.1% BSA in HBSS (Gibco, Grand Island, NY). Cell viability was assessed with Trypan blue and showed >85% viability. Afterward, cells were sent to the JHMI Transcriptomics and Deep Sequencing Core. The total cells were loaded onto the 10X Genomics chromium controller to generate single-cell barcoded droplets (GEMs) according to the manufacturer’s protocol with the 10× single-cell 3′ v2 chemistry, aiming at 10,000 cells per channel. The resulting libraries were sequenced on an Illumina NovaSeq S2 100 cycle (San Diego, CA). CellRanger was used to perform sample demultiplexing, barcode processing, and single-cell gene counting (Alignment, Barcoding and UMI Count) at the JHMI Transcriptomics and Deep Sequencing Core.

Downstream analysis steps were performed with Seurat. Cells were initially filtered to have >200 and <8000 detected genes, as well as less than 14% mitochondrial transcripts. CellCycleScoring function was used for regression out cell cycle impact. Dimensional reductions by means of uniform manifold approximation and projection (UMAP) were performed using Seurat. Pathway activity scores were generated with the AddModuleScore function of Seurat utilizing validated gene lists from KEGG pathways or GO term. Monocle2 was used for pseudotime trajectory analysis. Data from the intact calvaria was used in Fig. 2, and also compiled with bone defect time points (days 7 and 28) and used in Fig. 3.

### Spatial sequencing

In this study, our previously generated dataset was re-analyzed [20]. Spatial transcriptomics was performed using Visium Spatial Gene Expression system (10X Genomics) as previously described [20]. Calvaria from new-born pups (P0) were collected and fresh frozen, then embedded in OCT. The samples were cryo-sectioned at −30 °C at a thickness of 12 μm and placed onto the capturing windows of the Visium slides. Both tissue optimization process and gene expression assay were performed as per manufacturer’s instructions. Samples were fixed by methanol at −20 °C and stained with H&E. A tile scan of the capturing areas was generated using Leica DM6 B microscope (Leica Microsystems Inc.). After tissue the optimal permeabilization time was determined, the cDNA library was generated. Samples were then sequenced using an Illumina HiSeq system and the alignment and demultiplexing were conducted using the SpaceRanger pipeline.

### Isolation and culture of mouse calvarial suture cells

Wild-type (WT, C57BL/6J) or *Mkx*^fl/fl^ mice at indicated age (Supplementary Table S11) were used for suture cell isolation. After removing the periosteum and dura, the sagittal and coronal sutures were excised along with ∼0.5 mm of abutting parietal bone and frontal bone on each side. The suture tissues were minced and transferred into 10-cm petri dishes. All cells were cultured in Dulbecco’s Modified Eagle Medium (DMEM, Gibco, Grand Island, NY) supplemented with 15% fetal bovine serum (FBS, Gibco), 100 U/ml penicillin and 100 µg/ml streptomycin (Gibco) in a humidified incubator with 5% CO_2_ at 37 °C. After 3–5 d in culture, calvarial suture cells had migrated from the minced tissues and were passaged for experimental use. Medium was changed every 3 d. The cells of passage 2 or 3 were used for all experiments.

### siRNA knockdown

For siRNA knockdown experiments, suture mesenchymal cells from C57BL/6J mice were used. In brief, C57BL/6J mice were euthanized, and the coronal and sagittal sutures of the mice were micro-dissected as aforementioned. The calvarial sutures were minced and cultured in 10-cm petri dishes to allow the migration of suture cells. After being cultured for 3–4 days, the cells were subjected to fluorescence-activated cell sorting (FACS). The cells were incubated with APC anti-CD31, APC anti-CD45 and APC anti-Ter119 antibodies (details in Supplementary Table S12). To assess the cell viability, propidium iodide (BD Pharmingen, San Diego, CA) was added to the cells. FACS was performed using Beckman MoFlo (Beckman, Indianapolis, IN), and the data were analyzed using FlowJo software (BD, Ashland, OR). The lineage negative cells (CD31^−^CD45^−^Ter119^−^) were isolated and cultured in α-MEM, 15% FBS, 1% penicillin/streptomycin. After the cells were attached, the cells were used for further experiments. *Mkx* siRNA (Cat# s102302 and s102300) and negative control siRNA (Cat# 4390843) were obtained from Thermo Fisher Scientific. TransIT-LT1 Transfection Reagent (Mirus Bio, Madison, WI) was used as described by the manufacturer. Briefly, cells were plated at an initial density of 2 × 10^5^ cells/ml in 6-well cell culture plates and incubated for 24 h. Cells were then transfected with TransIT-LT1 Reagent-plasmid DNA complex containing 250 μl of Opti-MEM Reduced-Serum Medium (Gibco), 2.5 μg siRNA and 7.5 μl TransIT-LT1 Reagent and incubated for 48 h. Knockdown efficiency was confirmed by real-time polymerase chain reaction (qPCR). N = 3 replicates performed.

### qRT-PCR

Total RNA was extracted from cultured cells of equal passage number and density using TRIzol Reagent (Invitrogen, Carlsbad, CA) according to the manufacturer’s instructions. 1 μg of total RNA was used for reverse transcription with iScript cDNA synthesis kit (Bio-Rad). Real-time PCR was performed using SYBR Green PCR Master Mix (Thermo Scientific, Waltham, MA). Real-time PCR was performed using SYBR Green PCR Master Mix (Thermo Scientific, Waltham, MA). Relative gene expression was calculated using a 2^−^^ΔΔCt^ method by normalization with *Gapdh*. See Supplementary Table S13 for primer details.

### Bulk RNA sequencing and data analysis

Gene expression was detected by total RNA sequencing using the Illumina NextSeq 500 platform (Illumina, San Diego, CA). Briefly, total RNA was extracted from suture cells with or without *Mkx* KD by Trizol (Life Technologies Corporation, Gaithersburg, MD) and three independent RNA samples were prepared. The total RNA samples were sent to the JHMI Deep Sequencing and Microarray core for sequencing. Data analysis was performed using software packages including CLC Genomics Server and Workbench (RRID: SCR_017396 and RRID: SCR_011853), Partek Genomics Suite (RRID: SCR_011860), Spotfire DecisionSite with Functional Genomics (RRID: SCR_008858), and QIAGEN Ingenuity Pathway Analysis (IPA, RRID: SCR_008653). Kyoto Encyclopedia of Genes and Genomes (KEGG) and Gene Ontology (GO) enrichment analysis of differential expression genes (DEGs) were performed in Database for Annotation, Visualization, and Integrated Discovery (DAVID) bioinformatics software.

### In vivo knockout of *Mkx*

In vivo regional knockout of *Mkx* was achieved using virus transduced Cre recombinase as previously described [21]. Cre recombinase adenovirus (Ad-Cre, Cat. No: 1700) and control adenovirus (Ad-GFP, Cat. No: 1060) were purchased from Vector Biolabs (Malvern, PA). In brief, 2 d before calvarial defect surgery, at least 10^8^ CFU of Ad-Cre or Ad-GFP was injected into the center of calvaria midline each *Mkx*^fl/fl^ mouse subcutaneously (N = 5 male mice per group). Then calvarial defect procedures were performed as described above. An additional dose of adenovirus was administered to each mouse 2 d after the calvarial defect surgery. 28 d post-surgery, the mice were sacrificed and the calvaria were dissected and subjected to further analyses as above.

### Osteogenic differentiation assays

Cells were seeded into culture plates and cultured until 90% confluency. Then, osteogenic differentiation medium was added, composed of α-MEM, 15% FBS, 1% penicillin/streptomycin with 100 nM dexamethasone, 2 mM β-glycerophosphate (Sigma-Aldrich), and 50 μM ascorbic acid (Sigma-Aldrich). The medium was changed every 3 d. For alkaline phosphatase (ALP) staining, after 7 d of osteogenic differentiation, the cells were fixed with 4% PFA at RT for 10 min and washed well with PBS. Then, the cells were stained with BCIP/NBT staining solution (Sigma-Aldrich) at 37 °C for 30 min. Then, the wells were washed thoroughly with dH_2_O and imaged. Quantitative analysis of the intensity of ALP stain was assessed by dissolving stained cells in 10% cetylpyridinium chloride and then quantified by absorbance at 562 nm. In order to detect mineralization, cells were stained with 1% alizarin red S (Sigma-Aldrich) at 21 d of differentiation. The wells were imaged and calcium precipitate was dissolved by 0.1 N sodium hydroxide and quantified by absorbance at 548 nm. N = 3 experimental replicates.

### Mechanical stretching assays

Mechanical loading of the suture cells was conducted using the Cytostretcher system (Curi Bio, Seattle, WA). In brief, the cells were seeded into 6-mm chambers which were pre-coated with 0.2% gelatin as per manufacturer’s instruction. After the cells are attached to the chamber overnight, the chambers were loaded onto the Cytostretcher and subjected to 5% biaxial strain, at 0.5 Hz for 2 h under standard cell-cultural conditions. The mechanical loading to the cells was repeated for 3 d before further experiments. N = 3 experimental replicates performed.

### Statistical and power analyses

All experiments were performed and analyzed in a blinded fashion. Quantitative data are represented as mean ± 1 SD. Statistical analyses were performed using GraphPad Prism (RRID: SCR_002798, Version 7.0) or R package ggpubr. Sample size calculation was performed for experiments presented in Fig. 5 as based on an anticipated effect size of larger than 4 using our in vitro mineralization data in *Mkx*^fl/fl^ suture cells. For this scenario, with five samples per group, a two-sample *t*-test would provide 80% power to detect effect size of at least 2.0 assuming a two-tailed 0.05 level of significance. Unpaired two-tailed Student t-test was used for a two-sample comparison. One-way variance (ANOVA) followed by Tukey’s multiple comparisons or Kruskal–Wallis test was performed for multiple groups comparison. *P < 0.05 and **P < 0.01 were considered significant.

### Supplementary information

Supplementary figures and tables

Supplementary File S1

Supplementary File S2

Supplementary File S3

Supplementary File S4

## Data Availability

Single-cell RNA-seq and Bulk RNA-seq data are freely available within the NCBI GEO database GSE245094, GSE246238. The following dataset used in this study was previously published: GSE174313.
