# Supplementary material for: The Mohawk homeobox gene represents a marker and osteo-inhibitory factor in calvarial suture osteoprogenitor cells
Source: Cell Death Dis. 2024 Jun 17;15(6):420. doi: 10.1038/s41419-024-06813-4 (PMC11183145; doi:10.1038/s41419-024-06813-4)
Supplement: Supplementary file 1 — Supplementary figures and tables [file 41419_2024_6813_MOESM1_ESM.docx]

**Supplementary Materials**

**Supplementary Figure S1.** Generation and characterization of the *Mkx^CG^* allele.

**Supplementary Figure S2.** Overlap of tdT reporter activity and *Mkx* immunostaining.

**Supplementary Figure S3.** *Mkx* is expressed within the stem cell rich central region of the sagittal suture.

**Supplementary Figure S4.** scRNA seq sample preparation and feature genes for clustering.

**Supplementary Figure S5.** Fractions of different populations of cells over time by scRNA-sequencing.

**Supplementary Figure S6.** *Mkx*-eGFP and *Mk*x-tdT co-localization in healing calvarial bone.

**Supplementary Figure S7.** PCA of total RNA sequencing with or without Mkx knockdown in calvarial suture cells.

**Supplementary Table S1.** Top 20 GO terms upregulated in Subcluster 1 of Suture cells (Fig. 2G) by p-value using single-cell RNA sequencing.

**Supplementary Table S2.** Top 20 GO terms upregulated in Subcluster 2 of Suture cells (Fig. 2G) by p-value using single-cell RNA sequencing.

**Supplementary Table S3.** Top 20 GO terms upregulated in Subcluster 3 of Suture cells (Fig. 2G) by p-value using single-cell RNA sequencing.

**Supplementary Table S4.** Top 20 GO terms upregulated in Subcluster 4 of Suture cells (Fig. 2G) by p-value using single-cell RNA sequencing.

**Supplementary Table S5.** Top 20 GO terms upregulated in tdT^+^ cells D7 vs intact (Fig. 3G) by p-value using single cell RNA sequencing.

**Supplementary Table S6.** Top 20 GO terms upregulated in si*Mkx* vs control by p-value using total RNA sequencing.

**Supplementary Table S7.** Top 20 GO terms downregulated in si*Mkx* vs control by p-value using total RNA sequencing.

**Supplementary Table S8.** Top 20 KEGG pathway upregulated in si*Mkx* vs control by p-value using total RNA sequencing.

**Supplementary Table S9.** Top 20 KEGG pathway downregulated in si*Mkx* vs control by p-value using total RNA sequencing.

**Supplementary Table S10.** Mouse strains used in the study.

**Supplementary Table S11.** Table of detailed animal information.

**Supplementary Table S12.** List of antibodies used.

**Supplementary Table S13.** PCR primers used

**Supplementary File S1.** Feature genes for scRNA-seq clustering (Fig.2A, B, and E).

**Supplementary File S2.** Gene lists used in module score (Fig. 2H, Fig. 3G).

**Supplementary File S3.** Top 200 DEGs upregulated and downregulated among *Mkx* KD calvarial suture cells using total RNA sequencing (Fig. 6A).

**Supplementary File S4.** Gene lists used in heatmaps (Fig. 6E-J).

**Supplementary Figure S1**


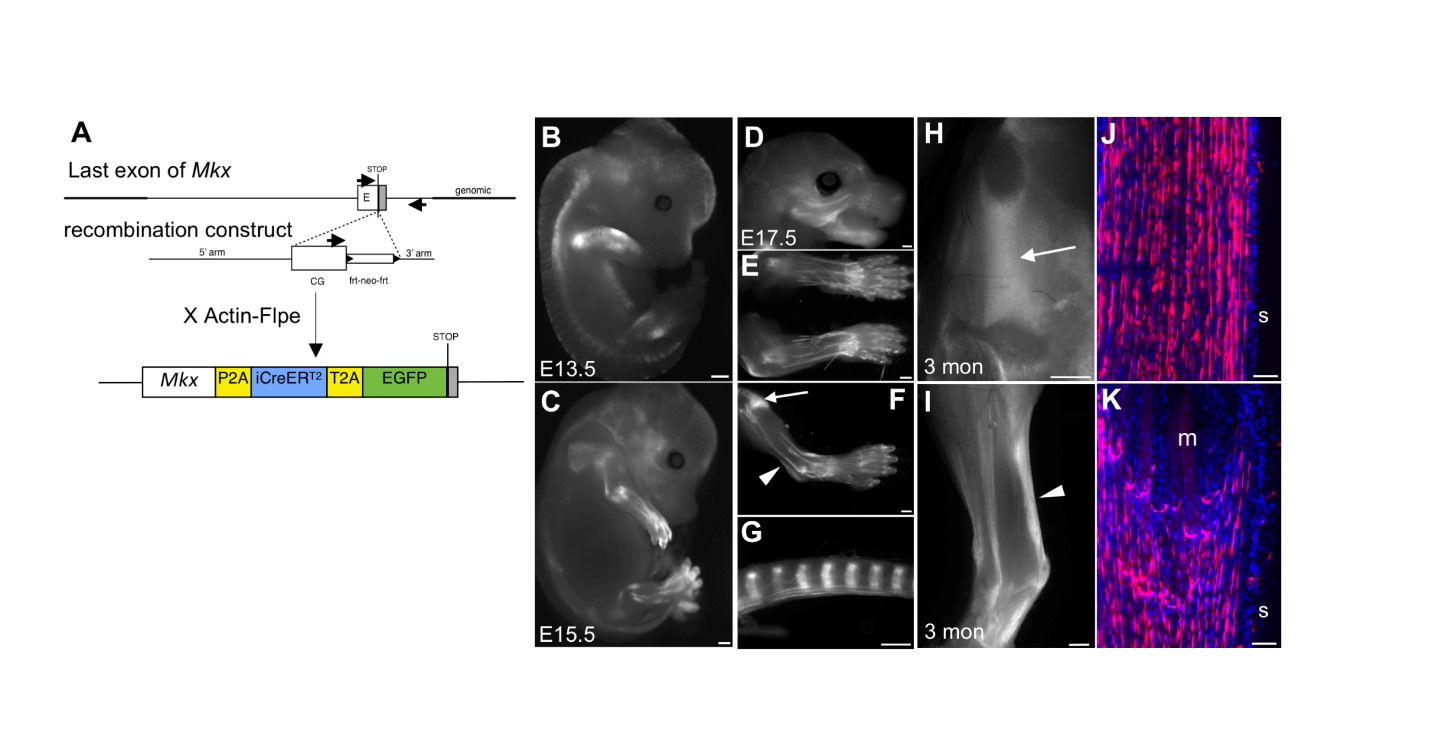


**Supplementary Fig. S1. Generation and characterization of the *Mkx^CG^* allele.** (**A**) Diagrams of the *Mkx* locus (top), the recombination construct (Middle), and the *Mkx^CG^* allele (bottom) after removal of the frt-neo-frt segment by crossing to the Actin-Flpe mouse. Recombinant DNA components within the CG cassette are labeled. (**B, C**) Whole-mount fluorescent images of a E13.5 *Mkx^CG^; R26R^tdT^* embryo received tamoxifen on E11.5 (**B**), and a E15.5 embryo received tamoxifen on E13.5 (**C**). (**D-G**) Whole-mount fluorescent image of select body parts of a *Mkx^CG^; R26R^tdT^* E17.5 embryo received tamoxifen at E15.5; **D**, head; **E**, forelimbs; **F**, hindlimb; **G**, tail. Arrows: Achilles tendon, arrowheads: Patellar tendon, and s: tendon sheath. Scale bars: 100 μm.

**Supplementary Figure S2.**


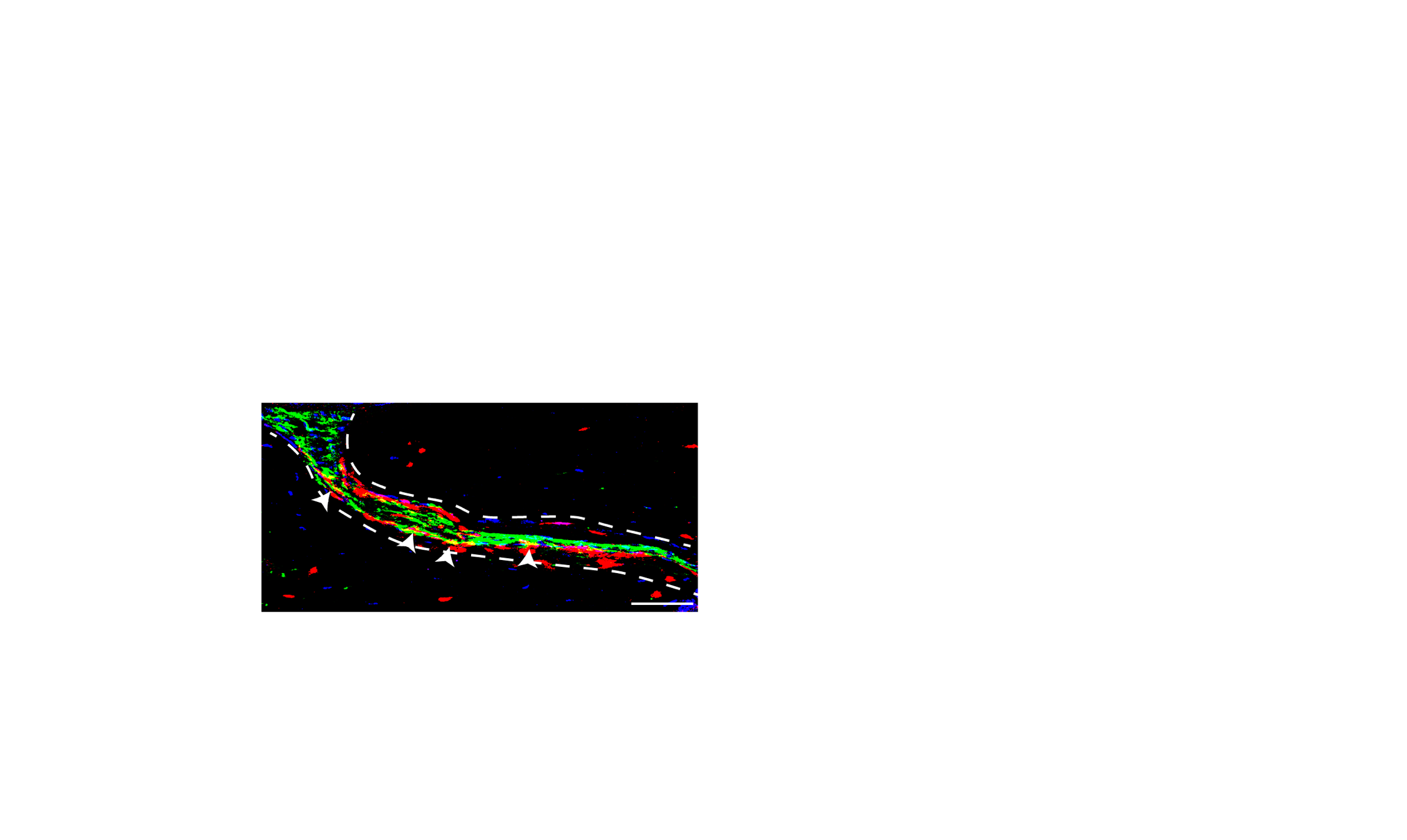


**Supplementary Fig. S2. Overlap of tdT reporter activity and *Mkx* immunostaining.** Fluorescent IHC of 16 week-old male *Mkx*^CG^;R26R^tdT^ mouse sagittal suture. The mouse was injected (i.p.) with TM and euthanized 14 days later. Sections of 12 μm were made and stained with anti-Mkx antibody. Mkx was shown in Green and tdT was shown in red. White arrows highlight the overlap between Mkx and tdT. Scale bar: 50 μm.

**Supplementary Figure S3**


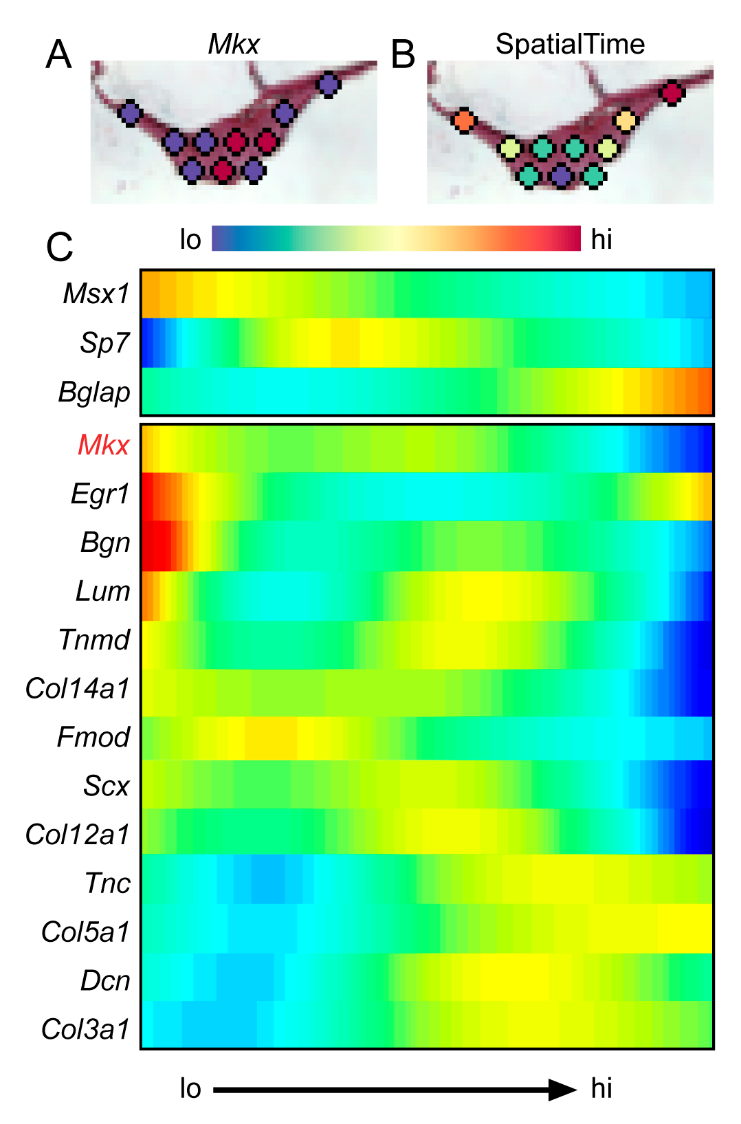


**Supplementary Fig. S3. *Mkx* is expressed within the stem cell rich central region of the sagittal suture.** A previously generated dataset was re-analyzed for this study (20). P 0 calvariae from mice were used for the spatial gene expression study. As a result, 1,670 spatial spots were detected by the technique across the tissue and each spatial spot contained 3,500 genes on average and 10,500 unique mRNA molecules. (**A**) Spatial feature plot of *Mkx* within the sagittal suture. (**B**) SpatialTime analysis of the sagittal suture reflecting the relative position of each spatial spot relative to the central suture (low) and osteogenic front (high). (**C**) Expression of tendon associated genes across SpatialTime.

**Supplementary Figure S4**


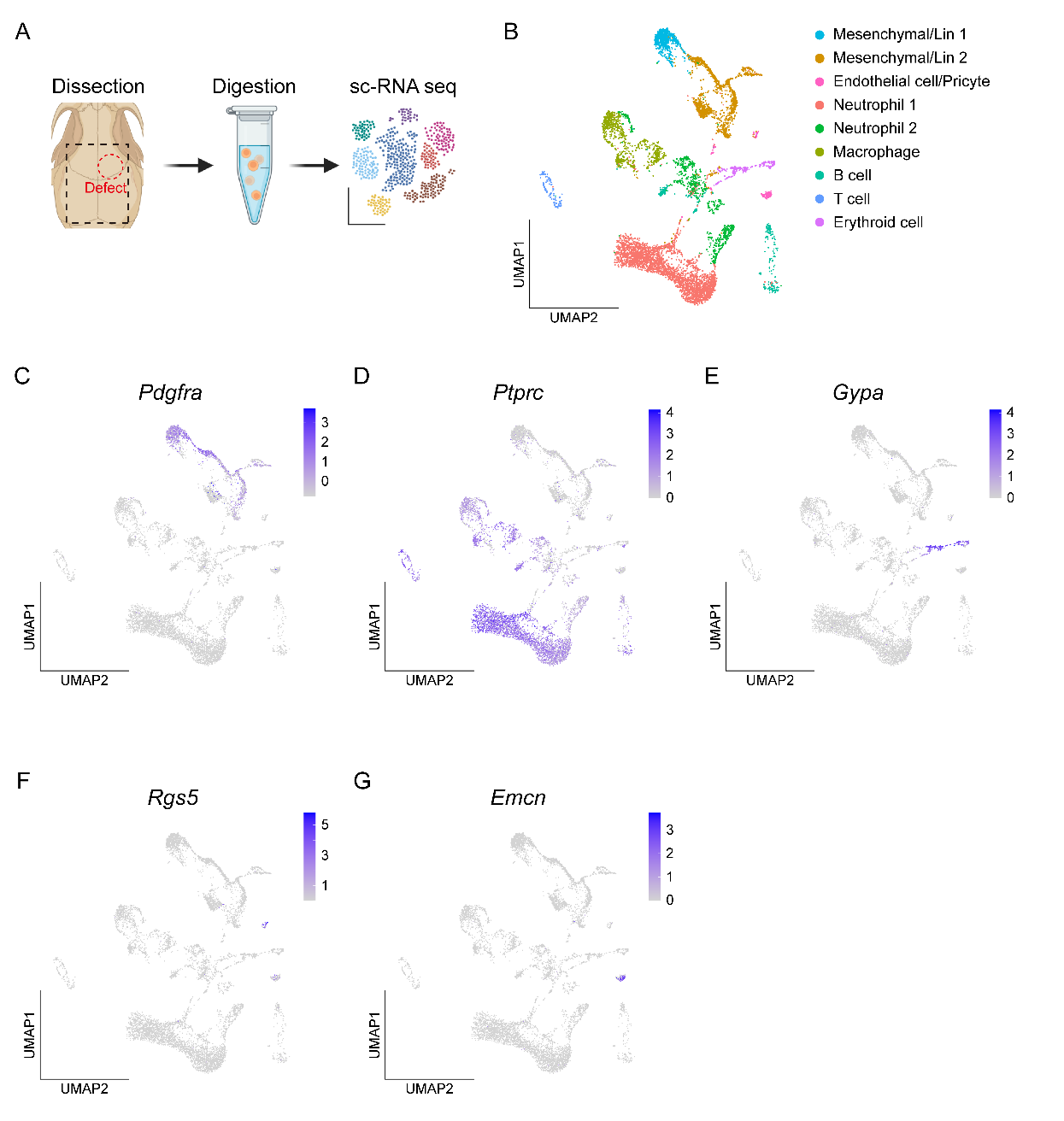


**Supplementary Fig. S4. scRNA seq sample preparation and feature genes for clustering.** (**A**) Schematic of sample preparation for scRNA-seq. Black dashed box shows the dissection area, including frontal bone, parietal bone, coronal suture, and sagittal suture. Red dashed circle shows defect area. (**B**) Uniform manifold approximation and projection (UMAP) plot of total cells isolated from intact calvarial bone. (**C-D**) Feature plot of the maker genes for mesenchymal maker (**C**) *Pdgfra*, hematopoietic cell marker (**D**) *Ptprc*, (**E**) *Gypa*, and endothelial/pericytes maker (**F**) *Rgs5* and (**G**) *Emcn*.

**Supplementary Figure S5**


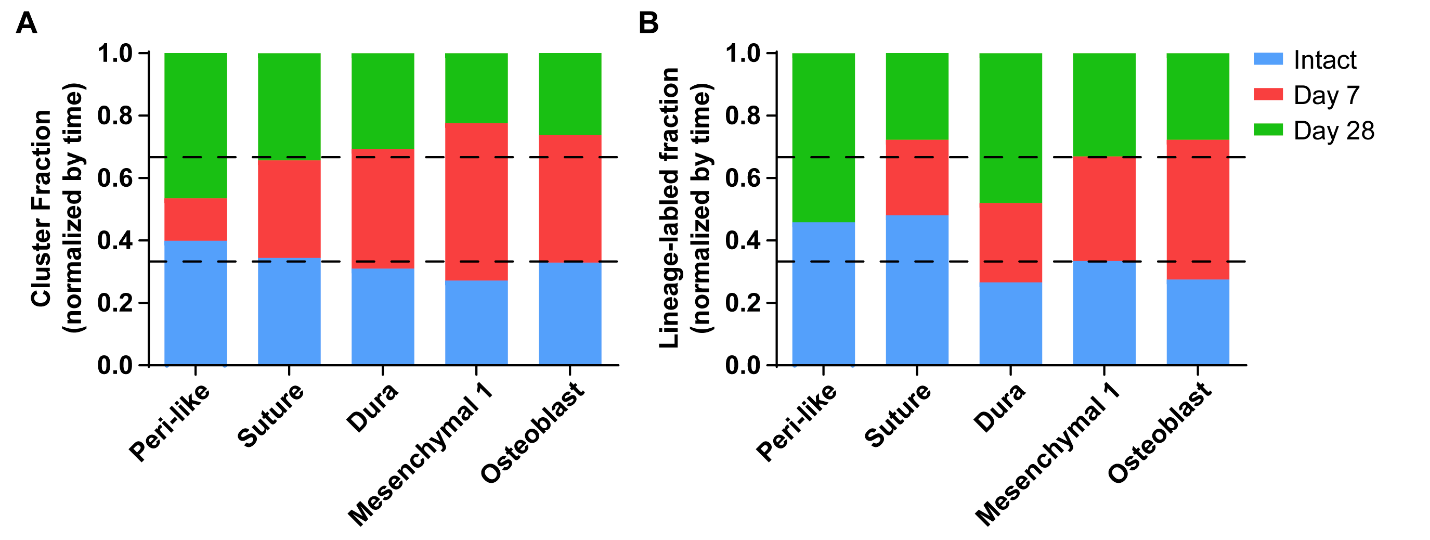


**Supplementary Fig. S5. Fractions of different populations of cells over time by scRNA-sequencing.** (**A**) Cell populations of different mesenchymal clusters at different timepoints. Cells from intact calvarial bone were shown in blue, Day 7 in red and Day 28 in green. (**B**) Fraction of tdT+ cells in different cell subclusters over time. The dashed lines represent 0.33 and 0.67.

**Supplementary Figure S6**

**
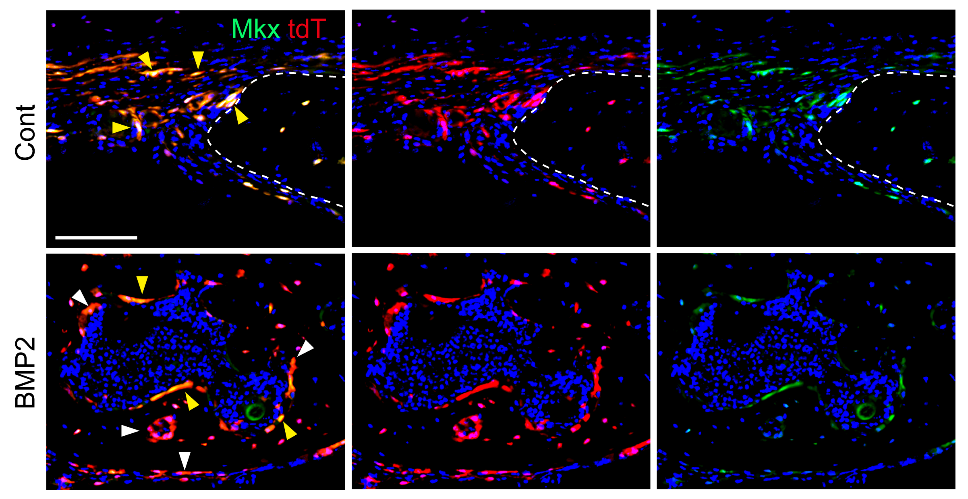
**

**Supplementary Fig. S6. *Mkx*-eGFP and *Mkx*-tdT reporter activity in healing calvarial bone**. Calvarial bone defect with or without BMP2 treatment at 28 d post-defect in *Mkx*^tdT^ reporter mice. Red: tdT reporter activity. Green: eGFP reporter activity. Blue: nuclear counterstain. Yellow arrowhead: dual reporter activity. White arrowhead: tdT reporter activity. The margins of original defect are indicated by dashed white. Scale bar: 20 μm.

**Supplementary Figure S7**

**
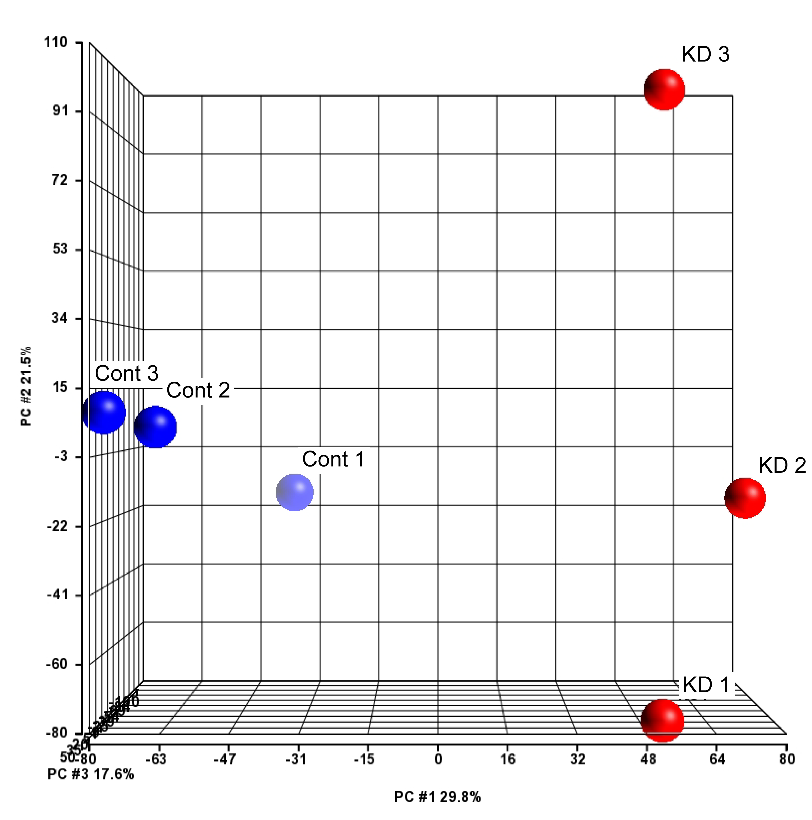
**

**Supplementary Fig. S7. PCA of total RNA sequencing with or without *Mkx* knockdown in calvarial suture cells.** Mouse calvarial suture cells underwent scramble or siRNA *Mkx* gene knockdown for 48 h. 16,478 protein-coding genes were identified for PCA. Blue dots represent control cells, while red dots show cells after *Mkx* KD. N=3 biological replicates.

| **Supplementary Table S1.** Top 20 GO terms enriched in Subcluster 1 of Suture cells (Fig. 2G) by p-value using single-cell RNA sequencing. | | |
| --- | --- | --- |
| **Term** | **Count** | **p-value** |
| Blood vessel development | 53 | 2.07509E-19 |
| Vasculature development | 54 | 3.02115E-19 |
| Blood vessel morphogenesis | 46 | 6.99158E-17 |
| Positive regulation of locomotion | 44 | 8.10803E-17 |
| Positive regulation of cellular component movement | 43 | 4.841E-16 |
| Positive regulation of cell motility | 42 | 9.17566E-16 |
| Positive regulation of cell migration | 40 | 6.30504E-15 |
| Angiogenesis | 39 | 9.3711E-15 |
| Response to interferon-beta | 15 | 1.88987E-14 |
| Extracellular matrix organization | 29 | 2.92244E-14 |
| Extracellular structure organization | 29 | 3.18016E-14 |
| External encapsulating structure organization | 29 | 3.18016E-14 |
| Leukocyte migration | 30 | 4.09721E-13 |
| Regulation of leukocyte migration | 23 | 1.43438E-12 |
| Response to growth factor | 39 | 2.41264E-12 |
| Cellular response to growth factor stimulus | 38 | 5.33232E-12 |
| Cellular response to interferon-beta | 12 | 9.58228E-12 |
| Cytokine-mediated signaling pathway | 28 | 2.38599E-11 |
| Ameboidal-type cell migration | 30 | 4.9128E-11 |
| Wound healing | 27 | 5.61104E-11 |

| **Supplementary Table S2.** Top 20 GO terms enriched in Subcluster 2 of Suture cells (Fig. 2G) by p-value using single-cell RNA sequencing. | | |
| --- | --- | --- |
| **Term** | **Count** | **p-value** |
| Cartilage development | 19 | 2.7645E-14 |
| Extracellular matrix organization | 22 | 4.5231E-14 |
| Extracellular structure organization | 22 | 4.8356E-14 |
| External encapsulating structure organization | 22 | 4.8356E-14 |
| Connective tissue development | 21 | 8.8799E-14 |
| Ossification | 24 | 2.2938E-13 |
| Negative regulation of cell differentiation | 31 | 2.955E-13 |
| Skeletal system development | 27 | 3.0633E-13 |
| Negative regulation of cell population proliferation | 30 | 1.2098E-12 |
| Regulation of cellular response to growth factor stimulus | 19 | 1.2826E-11 |
| Angiogenesis | 24 | 9.9146E-11 |
| Response to growth factor | 26 | 1.2265E-10 |
| Respiratory system development | 17 | 2.2345E-10 |
| Bone development | 16 | 3.6444E-10 |
| Vasculature development | 28 | 3.7004E-10 |
| Cellular response to growth factor stimulus | 25 | 4.1386E-10 |
| Tissue morphogenesis | 26 | 4.1812E-10 |
| Epithelial cell proliferation | 21 | 4.7446E-10 |
| Blood vessel development | 27 | 4.493E-10 |
| Blood vessel morphogenesis | 25 | 8.3699E-10 |

| **Supplementary Table S3.** Top 20 GO terms enriched in Subcluster 3 of Suture cells (Fig. 2G) by p-value using single-cell RNA sequencing. | | |
| --- | --- | --- |
| **Term** | **Count** | **p-value** |
| Actin cytoskeleton organization | 76 | 1.32925E-29 |
| Ossification | 58 | 2.33493E-29 |
| Supramolecular fiber organization | 71 | 9.63004E-25 |
| Cell-substrate adhesion | 47 | 2.25573E-22 |
| Skeletal system development | 57 | 2.945E-22 |
| Blood vessel development | 64 | 8.27212E-20 |
| Vasculature development | 65 | 1.96084E-19 |
| Extracellular matrix organization | 40 | 4.06389E-19 |
| Actin filament organization | 48 | 4.16737E-19 |
| Extracellular structure organization | 40 | 4.57626E-19 |
| External encapsulating structure organization | 40 | 4.57626E-19 |
| Blood vessel morphogenesis | 58 | 7.6501E-19 |
| Connective tissue development | 38 | 1.1291E-18 |
| Tissue morphogenesis | 58 | 6.24875E-18 |
| Cell junction organization | 59 | 1.59048E-16 |
| Cell-matrix adhesion | 31 | 4.06324E-16 |
| Actin filament bundle organization | 28 | 4.9942E-16 |
| Chondrocyte differentiation | 23 | 6.18128E-16 |
| Morphogenesis of an epithelium | 50 | 6.97434E-16 |
| Response to growth factor | 53 | 7.5977E-16 |

| **Supplementary Table S4.** Top 20 GO terms enriced in Subcluster 4 of Suture cells (Fig. 2G) by p-value using single-cell RNA sequencing. | | |
| --- | --- | --- |
| **Term** | **Count** | **p-value** |
| Leukocyte migration | 48 | 1.14E-28 |
| Inflammatory response | 36 | 4.80E-27 |
| Myeloid leukocyte activation | 26 | 8.40E-27 |
| Cell chemotaxis | 23 | 6.42E-26 |
| Leukocyte chemotaxis | 46 | 6.58E-25 |
| Myeloid leukocyte migration | 46 | 8.13E-24 |
| Regulation of cell activation | 21 | 1.48E-22 |
| Regulation of cytokine production | 16 | 1.95E-22 |
| Regulation of leukocyte activation | 32 | 1.37E-21 |
| Leukocyte cell-cell adhesion | 31 | 1.33E-20 |
| Myeloid leukocyte mediated immunity | 15 | 7.48E-19 |
| Granulocyte migration | 31 | 1.29E-18 |
| Regulation of defense response | 9 | 6.16E-18 |
| Neutrophil migration | 11 | 1.86E-17 |
| Chemotaxis | 13 | 2.14E-17 |
| Lymphocyte proliferation | 7 | 1.22E-16 |
| Granulocyte activation | 6 | 1.39E-16 |
| Mononuclear cell proliferation | 7 | 1.61E-16 |
| Negative regulation of immune system process | 63 | 2.00E-16 |
| Negative regulation of leukocyte activation | 63 | 2.99E-16 |

| Supplementary Table S5. Top 20 GO terms upregulated in tdT+ cells D7 vs intact (Fig. 3G) by p-value using single cell RNA sequencing. | | |
| --- | --- | --- |
| Term | **Count** | **p-value** |
| Regulation of bone mineralization | 6 | 1.40E-06 |
| Biomineral tissue development | 6 | 7.67E-06 |
| Ossification | 8 | 3.93E-05 |
| Translation | 14 | 1.40E-04 |
| Collagen fibril organization | 5 | 5.24E-04 |
| Transport | 33 | 5.74E-04 |
| Chondrocyte differentiation | 5 | 9.86E-04 |
| Protein folding | 7 | 0.0015 |
| Osteoblast differentiation | 6 | 0.00474 |
| Protein transport | 14 | 0.004855 |
| SRP-dependent co-translational protein targeting to membrane | 3 | 0.005703 |
| Positive regulation of epithelial cell proliferation | 5 | 0.006205 |
| ER to Golgi vesicle-mediated transport | 5 | 0.006798 |
| Cartilage development | 5 | 0.008093 |
| Cell-cell adhesion | 7 | 0.010056 |
| Response to vitamin D | 3 | 0.010112 |
| Endoplasmic reticulum unfolded protein response | 4 | 0.011032 |
| Cell growth | 4 | 0.011669 |
| Cell adhesion | 11 | 0.019181 |
| Positive regulation of protein localization to nucleus | 3 | 0.020402 |

| Supplementary Table S6. Top 20 GO terms upregulated in si*Mkx* vs control by p-value using total RNA sequencing. | | |
| --- | --- | --- |
| Term | **Count** | **p-value** |
| Immune system process | 61 | 9.22E-09 |
| Cellular response to interferon-beta | 18 | 1.20E-08 |
| Defense response to virus | 35 | 9.81E-08 |
| Positive regulation of angiogenesis | 28 | 1.91E-07 |
| Negative regulation of type I interferon-mediated signaling pathway | 11 | 3.39E-07 |
| Negative regulation of viral genome replication | 15 | 3.59E-07 |
| Angiogenesis | 36 | 3.41E-06 |
| Extracellular matrix organization | 27 | 5.70E-06 |
| Cell adhesion | 59 | 1.19E-05 |
| Response to virus | 17 | 2.93E-05 |
| Positive regulation of peptidyl-serine phosphorylation | 18 | 3.89E-05 |
| Positive regulation of cell migration | 32 | 5.52E-05 |
| Cell migration | 33 | 8.10E-05 |
| Cellular response to interferon-alpha | 8 | 8.23E-05 |
| Positive regulation of ERK1 and ERK2 cascade | 29 | 9.07E-05 |
| Positive regulation of gene expression | 54 | 1.52E-04 |
| Positive regulation of peptidyl-tyrosine phosphorylation | 18 | 2.20E-04 |
| Response to interferon-gamma | 9 | 3.08E-04 |
| Cell redox homeostasis | 9 | 3.08E-04 |
| Innate immune response | 60 | 3.09E-04 |

| Supplementary Table S7. Top 20 GO terms downregulated in si*Mkx* vs control by p-value using total RNA sequencing. | | |
| --- | --- | --- |
| Term | **Count** | **p-value** |
| Cell migration | 47 | 6.36E-14 |
| Cell cycle | 79 | 1.06E-13 |
| Integrin-mediated signaling pathway | 26 | 2.37E-11 |
| Actin filament organization | 31 | 4.34E-11 |
| Cell division | 53 | 7.25E-11 |
| Actin cytoskeleton organization | 35 | 1.28E-10 |
| Endocytosis | 33 | 1.89E-09 |
| Cell migration | 40 | 5.71E-09 |
| Regulation of cell shape | 28 | 7.17E-09 |
| Cell adhesion | 63 | 1.56E-08 |
| Cytoskeleton organization | 23 | 2.48E-08 |
| Positive regulation of gene expression | 60 | 8.22E-08 |
| Positive regulation of gtpase activity | 37 | 2.05E-07 |
| Double-strand break repair via break-induced replication | 8 | 4.21E-07 |
| DNA replication initiation | 10 | 1.56E-06 |
| Signal transduction | 97 | 2.30E-06 |
| Cell-matrix adhesion | 18 | 3.74E-06 |
| Positive regulation of protein phosphorylation | 32 | 4.11E-06 |
| Mitotic cytokinesis | 13 | 4.78E-06 |
| Small gtpase mediated signal transduction | 18 | 8.73E-06 |

| Supplementary Table S8. Top 20 KEGG pathway upregulated in si*Mkx* vs control by p-value using total RNA sequencing. | | |
| --- | --- | --- |
| Term | **Count** | **p-value** |
| Epstein-Barr virus infection | 38 | 2.58E-07 |
| Fluid shear stress and atherosclerosis | 25 | 2.72E-05 |
| Proteoglycans in cancer | 30 | 5.77E-05 |
| MAPK signaling pathway | 38 | 7.71E-05 |
| Kaposi sarcoma-associated herpesvirus infection | 30 | 2.81E-04 |
| Phagosome | 26 | 2.94E-04 |
| Viral carcinogenesis | 29 | 8.94E-04 |
| Human cytomegalovirus infection | 31 | 1.19E-03 |
| Coronavirus disease - COVID-19 | 30 | 1.39E-03 |
| Pathways in cancer | 54 | 1.74E-03 |
| Influenza A | 23 | 1.87E-03 |
| PI3K-Akt signaling pathway | 39 | 1.93E-03 |
| Proteasome | 10 | 2.89E-03 |
| Rap1 signaling pathway | 26 | 3.10E-03 |
| Ferroptosis | 9 | 3.69E-03 |
| Cell adhesion molecules | 22 | 4.40E-03 |
| Hepatocellular carcinoma | 22 | 4.40E-03 |
| Antigen processing and presentation | 14 | 5.25E-03 |
| TNF signaling pathway | 16 | 6.33E-03 |
| Melanoma | 12 | 6.47E-03 |

| Supplementary Table S9. Top 20 KEGG pathway downregulated in si*Mkx* vs control by p-value using total RNA sequencing. | | |
| --- | --- | --- |
| Term | **Count** | **p-value** |
| Regulation of actin cytoskeleton | 39 | 4.06E-10 |
| Focal adhesion | 34 | 2.20E-08 |
| Cell cycle | 25 | 9.47E-08 |
| PI3K-Akt signaling pathway | 46 | 2.96E-07 |
| Amoebiasis | 22 | 4.12E-07 |
| Chemokine signaling pathway | 29 | 3.06E-06 |
| ECM-receptor interaction | 18 | 6.56E-06 |
| Pathways in cancer | 56 | 1.26E-05 |
| Rap1 signaling pathway | 29 | 2.48E-05 |
| Leukocyte transendothelial migration | 20 | 2.96E-05 |
| Adherens junction | 15 | 3.36E-05 |
| Tight junction | 24 | 5.88E-05 |
| Platelet activation | 20 | 5.99E-05 |
| Osteoclast differentiation | 20 | 9.31E-05 |
| DNA replication | 10 | 1.01E-04 |
| Human T-cell leukemia virus 1 infection | 30 | 1.65E-04 |
| Yersinia infection | 20 | 1.73E-04 |
| Toxoplasmosis | 17 | 4.15E-04 |
| Human papillomavirus infection | 37 | 6.09E-04 |
| Endocytosis | 30 | 6.94E-04 |

| **Supplementary Table S10.** Mouse strains used in this study. | |
| --- | --- |
| **Mouse strain** | **Information** |
| **Mkx^CG^** | Created by Chen-Ming Fan Lab |
| **R26R^tdT/tdT^** | JAX Strain # 007909 |
| **R26R^iDTR/iDTR^** | JAX Strain # 007900 |
| **Mkx^tdT^** | Cross of *Mkx*^CG^ with R26R^tdT/tdT^ |
| **Mkx^tdT/iDTR^** | Cross of *Mkx*^tdT^ with R26R^iDTR/iDTR^ |
| **Mkx^fl/fl^** | Gifted by Han laboratory |
| **C57BL/6J** | JAX Strain # 000664 |

**Supplementary Table S11.** Table of detailed animal information.

|  | **Strain** | **Age** | **Gender** | **N** |
| --- | --- | --- | --- | --- |
| **Fig. 1** | *Mkx*^tdT^ | 15 weeks | Mixed | 3-5 |
| **Fig. 2, Fig. 3A-G** | *Mkx*^tdT^ | 8 weeks | Mixed | 3/timepoint |
| **Fig. 3H-O** | *Mkx*^tdT^ | 8-10 weeks | Mixed | 3 |
| **Fig. 4** | *Mkx*^tdT^ or *Mkx*^tdT/iDTR^ | 8-10 weeks | Mixed | 4-6 |
| **Fig. 5A, B** | C57BL/6J | P7 | Mixed | 5 |
| **Fig. 5C, D** | *Mkx*^fl/fl^ | P7 | Mixed | 5 |
| **Fig. 5E-M** | *Mkx*^fl/fl^ | 8-10 weeks | Mixed | 5 |
| **Fig. 6** | C57BL/6J | 8 weeks | Male | 5 |
| **Fig. 7** | C57BL/6J | 8 weeks | Male | 5 |

| **Supplementary Table S12.** List of antibodies used. | | | |
| --- | --- | --- | --- |
| **Antibody** | **Company** | **Catalog #** | **Use** |
| Mouse anti-Mkx | Novus Biologicals | NPB2-45863 | IF |
| Rabbit anti-Gli1 | Novus Biologicals | NPB1-78259 | IF |
| Rabbit anti-Axin2 | Abcam | ab107613 | IF |
| Rabbit anti-Ocn | Abcam | ab93876 | IF |
| Mouse anti-Tubb3 | Abcam | ab18207 | IF |
| Rabbit anti-CD31 | Abcam | ab28364 | IF |
| APC anti-mouse CD31 | Biolegend | 102409 | FC |
| APC anti-mouse CD45 | Biolegend | 157605 | FC |
| APC anti-mouse Ter119 | Biolegend | 116211 | FC |
| Goat anti-Mouse AF488 | Abcam | ab150117 | IF |
| Goat anti-Rabbit AF488 | Abcam | ab150077 | IF |
| Goat anti-Rabbit DyLight 594 | Vector Laboratories | DI-1594 | IF |
| IF: Immunofluorescent staining. FC: Flow cytometry | | | |

| **Supplementary Table S13.** PCR primers used. | | |
| --- | --- | --- |
| **Gene** | **Forward Primer (5’-3’)** | **Reverse Primer (5’-3’)** |
| **Alpl** | CCAGAAAGACACCTTGACTGTGG | TCTTGTCCGTGTCGCTCACCAT |
| **Col1a1** | CCTCAGGGTATTGCTGGACAAC | CAGAAGGACCTTGTTTGCCAGG |
| **Col3a1** | GACCAAAAGGTGATGCTGGACAG | CAAGACCTCGTGCTCCAGTTAG |
| **Fmod** | CCAACACCTTCAACTCCAGCAG | GTGCAGAAGCTGCTGATGGAGA |
| **Gapdh** | CATCACTGCCACCCAGAAGACTG | ATGCCAGTGAGCTTCCCGTTCAG |
| **Runx2** | CCTGAACTCTGCACCAAGTCCT | TCATCTGGCTCAGATAGGAGGG |
| **Scx** | ACCGCACCAACAGCGTGAACAC | CAGCACATTGCCCAGGTGAGAA |
| **Sox5** | CGCCAGATGAAAGAGCAACTCAG | TGAGTCAGGCTCTCCAGTGTTG |
| **Sox6** | GCATAAGTGACCGTTTTGGCAGG | GGCATCTTTGCTCCAGGTGACA |
| **Sp7** | GGCTTTTCTGCGGCAAGAGGTT | CGCTGATGTTTGCTCAAGTGGTC |
| **Tnmd** | AAGCAAGCGAGGAAGACCTTCC | TTCACAGACACGGCGGCAGTAA |
| **Mkx** | GGAAGGTAAGGCATAAGCGAC | GGTTGTCACGGTGCTTGTAAAG |
